# Transcriptomic comparison of human and mouse brain microvessels

**DOI:** 10.1038/s41598-020-69096-7

**Published:** 2020-07-23

**Authors:** Hannah W. Song, Koji L. Foreman, Benjamin D. Gastfriend, John S. Kuo, Sean P. Palecek, Eric V. Shusta

**Affiliations:** 1 Department of Chemical and Biological Engineering, University of Wisconsin-Madison, 1415 Engineering Dr., Madison, WI 53706 USA; 2 Department of Neurosurgery and Mulva Clinic for the Neurosciences, Dell Medical School, University of Texas at Austin, Austin, TX USA; 3 Department of Neurological Surgery, University of Wisconsin-Madison, Madison, WI USA; 4 Present Address: Wallace H. Coulter Department of Biomedical Engineering, Georgia Institute of Technology, Atlanta, GA USA

**Keywords:** Blood-brain barrier, Genetics of the nervous system

## Abstract

The brain vasculature maintains brain homeostasis by tightly regulating ionic, molecular, and cellular transport between the blood and the brain parenchyma. These blood–brain barrier (BBB) properties are impediments to brain drug delivery, and brain vascular dysfunction accompanies many neurological disorders. The molecular constituents of brain microvascular endothelial cells (BMECs) and pericytes, which share a basement membrane and comprise the microvessel structure, remain incompletely characterized, particularly in humans. To improve the molecular database of these cell types, we performed RNA sequencing on brain microvessel preparations isolated from snap-frozen human and mouse tissues by laser capture microdissection (LCM). The resulting transcriptome datasets from LCM microvessels were enriched in known brain endothelial and pericyte markers, and global comparison identified previously unknown microvessel-enriched genes. We used these datasets to identify mouse-human species differences in microvessel-associated gene expression that may have relevance to BBB regulation and drug delivery. Further, by comparison of human LCM microvessel data with existing human BMEC transcriptomic datasets, we identified novel putative markers of human brain pericytes. Together, these data improve the molecular definition of BMECs and brain pericytes, and are a resource for rational development of new brain-penetrant therapeutics and for advancing understanding of brain vascular function and dysfunction.

## Introduction

The blood–brain barrier (BBB) regulates blood flow, supplies the brain with nutrients, and facilitates clearance of a variety of substances. The BBB is comprised of brain microvascular endothelial cells (BMECs), the principal barrier-forming cell. BMECs are also intimately associated with brain pericytes, mural cells that line the outside of microvessels and are linked to endothelial cells by a shared vascular basement membrane^1^. The BBB is required to maintain brain homeostasis, but also prevents clinically relevant doses of many therapeutics from entering the brain^2,3^. Brain vascular dysfunction plays a role in several neurological disorders, including some with cell-autonomous defects in BMEC or pericyte function^4–7^. Due to its role in neurological disorders and important implications for brain drug delivery, the brain vasculature has been the subject of intense research, often focused on identifying mechanisms underlying its unique behavior. Our understanding of brain vascular development, function, dysfunction, and molecular constituents, however, has been advanced largely by mouse models. The scarcity of human brain tissue and low abundance of brain vascular cells has limited molecular profiling of the human brain vasculature. Improved molecular understanding of human BMECs and pericytes could aid in the development of new BBB-penetrant therapeutics and advance new hypotheses about mechanisms of brain vascular dysfunction in disease.

Mouse brain vascular cells have previously been isolated and transcriptionally profiled^8–12^. For example, fluorescence-activated cell sorting (FACS) has been used to isolate brain endothelial cells from *Tie2*-GFP mice, and microarray analysis identified transcripts that were enriched or depleted in brain endothelial cells compared to lung and liver endothelial cells^8^. Full transcriptomic datasets of mouse brain endothelial cells and pericytes have also been obtained by performing RNA sequencing (RNA-seq) after cell isolation by FACS or immunopanning methods^11,13^. Similarly, bulk and single cell RNA-seq of endothelial cells in mice have also been used to identify transcription factors and β-catenin-regulated genes related to blood–brain barrier specification during the developmental timecourse^12^. Recently, single cell RNA-seq has also been used to improve the transcriptomic definition of mouse brain pericytes and identify positional variation in mural and endothelial cell gene expression^9,10^. While the mouse data have been instrumental in advancing our understanding of BMECs and pericytes, there are numerous species-specific differences that have been identified between mouse and human brain including in solute carrier and efflux transporter expression^14–16^. Gene expression profiling in human brain samples has been much more limited and largely focused on non-vascular cell types, including neurons and astrocytes. For example, single cell RNA-seq of human adult and embryonic cortex samples has been used to identify regional, developmental, and species differences in neuronal and glial gene expression; however, the approaches employed yielded extremely small populations of endothelial cells and pericytes, impeding analysis of human brain microvessel transcriptomes^17–19^. Recently, brain microvessels isolated by centrifugation from two patient samples were subjected to RNA-seq to analyze expression of ATP binding cassette (*ABC*) transporters and solute carriers (*SLC*s)^20^, and brain endothelial cells isolated via immunopanning from two patient samples were also transcriptomically profiled^21^. A detailed, transcriptome-wide analysis of human brain microvessels, however, is currently lacking. Furthermore, the limited number of existing human brain vascular datasets motivates transcriptomic characterization of additional patient samples. Finally, a more complete understanding of mouse-human species differences may help inform choice of model systems for studying BBB biology or screening for brain penetrant therapeutics.

In this study, we performed RNA-seq on normal mouse and human brain microvessels isolated using laser capture microdissection (LCM). LCM allowed the isolation of brain microvessels from snap-frozen tissue sections, limiting possible transcriptional changes induced by techniques used in previous BBB transcriptomic analyses such as cell homogenization, sorting, immunopanning, or short periods of in vitro culture^22–24^. The isolated microvessels contained both BMEC and pericyte components and the transcriptomes were enriched in both BMEC and pericyte transcripts as expected. Data analysis allowed the identification of genes with microvessel-enriched expression despite patient-to-patient variability, identified species differences between mouse and human samples, and suggested putative novel human brain pericyte markers.

## Results

### Laser capture microdissection and RNA-sequencing of brain microvessels

**Figure 1:**
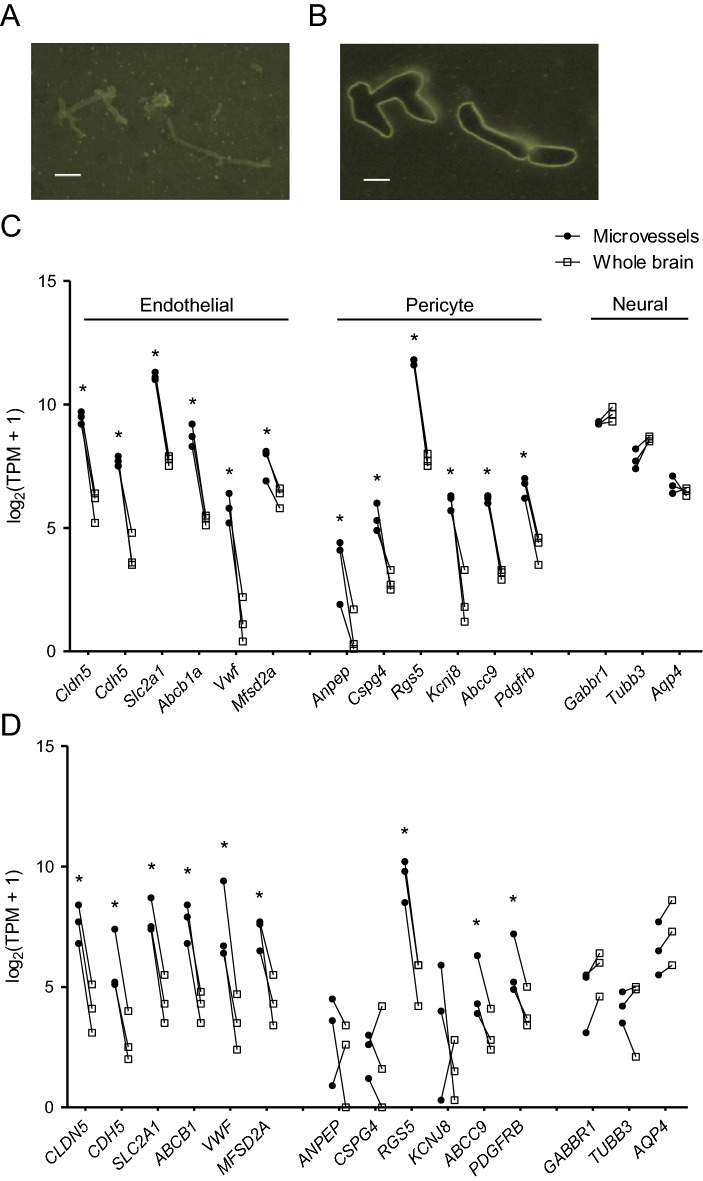
LCM of brain microvessels yields transcriptomic datasets enriched in endothelial and pericyte markers. (**A**,**B**) Representative lectin-stained mouse brain sections before (**A**) and after (**B**) LCM. Scale bars: 50 μm. (**C**,**D**) Transcript abundance [log_2_(TPM + 1)] of endothelial, pericyte, and neural genes in mouse (**C**) and human (**D**) whole brain and LCM microvessel samples. Lines connect datapoints from matched LCM microvessel and whole brain samples. *P < 0.05 versus whole brain, DESeq2 Wald test with Benjamini–Hochberg correction. Exact P-values are provided in Supplementary Table S4.

Normal brain tissue from three human patients undergoing neurosurgery was obtained and flash frozen immediately to preserve mRNA content. Similarly, brains were removed and flash frozen from three C57/BL6N mice. Tissue sections were first labeled with fluorescent lectins, which bind to the endothelial glycocaylx, to identify microvessels for capture (Fig. 1A). The lectin^+^ microvessels were manually outlined and captured for RNA isolation using an LCM system (Fig. 1B). To enrich samples for microvascular endothelial cells and associated pericytes while limiting the contribution of vascular smooth muscle cells, which vary in phenotype and gene expression from microvascular pericytes^10^, only vessels ≤ 10 μm in diameter were isolated. Given the spatial resolving constraints of LCM, it is impossible to isolate solely BMECs, and hence the isolated microvessels also contained microvessel-associated pericytes. Thus, this combination of BMECs and pericytes will be defined as “microvessels” throughout the manuscript. Microvessel fragments totaling roughly 2.5 mm^2^ and an equivalent area of whole brain tissue were isolated from each human and murine brain sample, yielding 0.8–5.5 ng of total RNA per sample (Supplementary Table S1; Supplementary Fig. S1).

RNA sequencing of the resulting LCM microvessel and whole brain RNA samples each yielded approximately 20 million reads, which were then aligned to the human and mouse genomes. The number of reads and alignment percentages for each sample are shown in Supplementary Table S1, and gene count data are provided in Supplementary Table S2. To validate the resulting datasets, we examined expression of a subset of known brain endothelial, pericyte, and neural (astrocyte and neuron) genes in LCM microvessels and whole brain. As expected, mouse LCM microvessels had increased expression of endothelial genes (*Cldn5*, *Cdh5*, *Slc2a1*, *Abcb1a*, *Vwf*, *Mfsd2a*) and pericyte genes (*Anpep*, *Cspg4*, *Rgs5*, *Kcnj8*, *Abcc9*, *Pdgfrb*), and no enrichment of neural genes (*Gabbr1*, *Tubb3*, *Aqp4*) compared to whole brain samples (Fig. 1C). In human LCM microvessels, we observed similar enrichment of endothelial genes (*CLDN5*, *CDH5, SLC2A1*, *ABCB1*, *VWF, MFSD2A*) and lack of enrichment of neural genes (*GABBR1, TUBB3, AQP4*). Compared to mouse, the human LCM microvessels had enrichment of *RGS5, ABCC9,* and *PDGFRB*, but lacked statistically significant enrichment of other pericyte markers, such as *ANPEP, CSPG4*, and *KCNJ8*, which may be a result of increased variability between human patient samples, lower pericyte numbers in human samples compared to mouse, or mouse-human species differences in pericyte gene expression (Fig. 1D).

**Figure 2:**
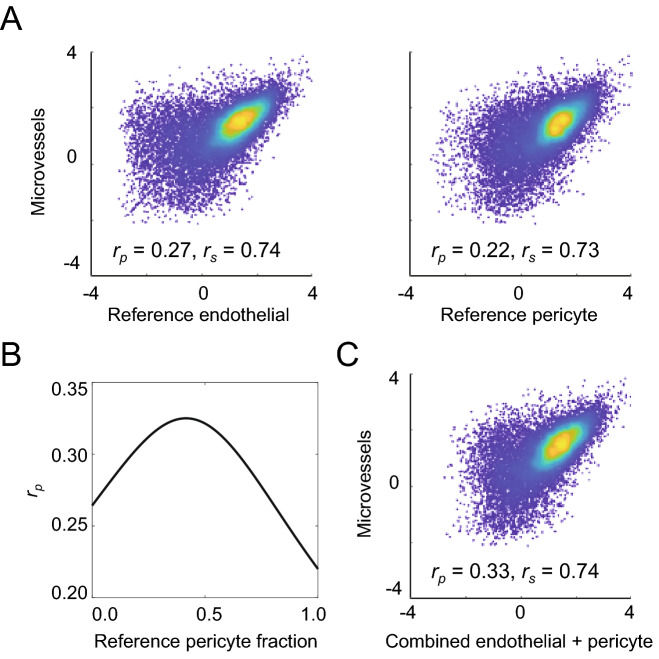
Comparison of mouse LCM microvessel with existing mouse BMEC and pericyte transcriptomic datasets. (**A**) Comparison of transcript abundances [log_10_(TPM)] in mouse LCM microvessels (y-axes; average of three biological replicates) versus the reference BMEC dataset or the reference pericyte dataset^10^. Each point represents one gene with nonzero expression in both LCM microvessel and reference endothelial or pericyte datasets. The Pearson (*r*_*p*_) and Spearman’s (*r*_*s*_) correlation coefficients are inset and were calculated on raw TPM values prior to removal of undetected transcripts and log-transformation. Pseudocoloring indicates relative population density. (**B**) Pearson correlation coefficient (*r*_*p*_) for the average of the biological replicates of mouse LCM microvessels calculated against reference datasets generated by combining brain pericyte and endothelial cell transcriptomes in different ratios (x-axis). (**C**) Comparison of transcript abundances [log_10_(TPM)] in mouse LCM microvessels (y-axis; average of three biological replicates) versus the optimal combined reference dataset (42% pericyte transcript and 58% BMEC transcript weighting). Each point represents one gene with nonzero expression in both LCM microvessel and the reference dataset. The Pearson (*r*_*p*_) and Spearman’s (*r*_*s*_) correlation coefficients are inset and were calculated on raw TPM values prior to removal of undetected transcripts and log-transformation. Pseudocoloring indicates relative population density.

To further examine the LCM microvessel datasets, we compared expression of all genes in the mouse samples to recent single cell RNA-seq datasets of FACS-purified mouse BMECs and pericytes (Supplementary Table S3)^10^. The Pearson correlation coefficient (*r*_*p*_) between LCM microvessels (average of three biological replicates) and the reference endothelial dataset was 0.27 (*P* < 0.001), while the correlation for the reference pericyte dataset was 0.22 (*P* < 0.001), indicating a positive, but poor correlation for both cell types (Fig. 2A). Spearman’s correlation coefficient (*r*_*s*_) between LCM microvessels and the reference endothelial dataset was 0.74, and for the reference pericyte dataset was 0.73, indicating similar ranking of genes despite poor correlation on absolute abundance (Fig. 2A). Since LCM microvessel datasets are derived from a mixture of cell types, largely BMECs and pericytes, these results were not surprising. In an effort to adjust for the presence of multiple cell types, we recalculated Pearson correlation coefficients between the LCM datasets and reference single cell RNA-seq datasets by mathematically combining different ratios of endothelial cell and pericyte datasets (see Methods for details). This analysis revealed an optimal Pearson correlation (*r*_*p*_ = 0.33, *P* < 0.001) for a reference dataset comprising approximately 42% pericyte-derived transcripts and 58% BMEC-derived (Fig. 2B, C), while the Spearman’s correlation coefficient (*r*_*s*_ = 0.74) did not increase over the endothelial-only dataset (Fig. 2C). The low Pearson correlation, and lack of improvement in Spearman’s correlation, is likely due to parenchymal cell contamination in the microvessel preparations and technical differences between single cell and bulk RNA-seq methodologies. Nonetheless, the non-monotonic relationship between Pearson correlation and ratio of endothelial cell and pericyte reference datasets supports the presence of both endothelial cells and pericytes in the LCM microvessel samples.

### Comparison of LCM and whole brain transcriptomes identifies microvessel-enriched genes

**Figure 3:**
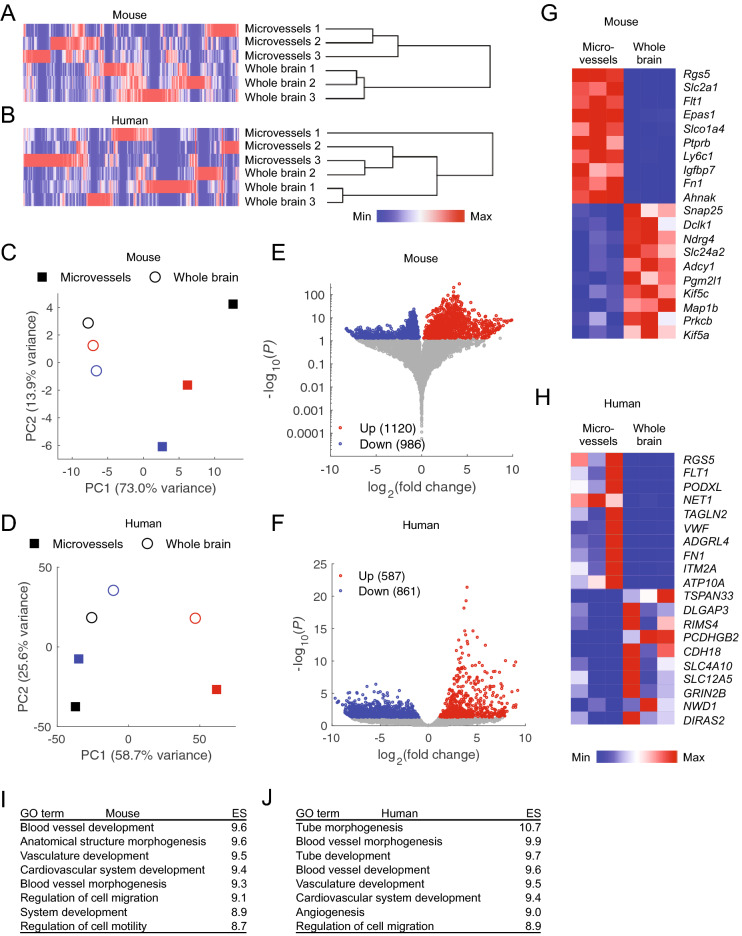
Differentially expressed genes in mouse and human LCM microvessels compared to whole brain. (**A**,**B**) Whole-transcriptome hierarchical clustering of mouse (**A**) and human (**B**) LCM microvessels and whole brain datasets. Color indicates expression that has been normalized within each gene (column). (**C**,**D**) Principal component analysis of mouse (**C**) and human (**D**) LCM microvessels and whole brain datasets. Data are plotted in the space of the first two principal components, with the percentage of variance explained by principal component 1 (PC1) and principal component 2 (PC2) shown in axis labels. Microvessel and whole brain datapoints of the same color are derived from matched samples. (**E**,**F**) Volcano plots illustrating genes differentially expressed between LCM microvessels and whole brain samples from mouse (**E**) and human (**F**). The number of LCM microvessel-enriched (Up) and depleted (Down) genes with adjusted P-values < 0.05 (from DESeq2) are shown in the legends. Full results of differential expression analysis are in Supplementary Table S4. (**G,H**) Heat maps illustrating transcript abundance in biological triplicates of LCM microvessels and whole brain for the 10 highest confidence LCM microvessel-enriched and the 10 highest confidence microvessel-depleted genes in mouse (**G**) and human (**H**). Color indicates expression that has been normalized within each gene (row). (**I,J**) Gene ontology (GO) terms for biological processes enriched in LCM microvessels compared to whole brain from mouse (**I**) and human (**J**). ES: enrichment score [− log_10_(*P*)].

We used hierarchical clustering and principal component analysis (PCA) to make transcriptome-wide, unbiased comparisons of LCM microvessels and whole brain. Mouse microvessel datasets clustered together and were distinct from whole brain datasets (Fig. 3A). Human datasets did not produce this expected clustering, with one whole brain dataset clustering more closely with a microvessel dataset from a different patient sample than with other whole brain datasets (Fig. 3B). In PCA, mouse microvessel and whole brain datasets also clustered together along principal component 1 (PC1; explaining 73% of the variance) (Fig. 3C), while weaker clustering along principal component 2 (PC2; explaining 26% of the variance) was observed for the human datasets (Fig. 3D). Together, these analyses suggest that patient-derived human datasets have more variability than mouse samples, potentially attributable to differences in genetic background, brain region, or underlying complications that required neurosurgical intervention.

We next identified genes differentially expressed in LCM microvessels compared to whole brain. This analysis revealed 1,120 genes enriched and 986 genes depleted in mouse LCM microvessels compared to whole brain (Fig. 3E; Supplementary Table S4). We similarly identified 587 enriched and 861 depleted genes in human LCM microvessels (Fig. 3F; Supplementary Table S4). Mouse microvessel-enriched genes included known pericyte and endothelial markers (*Rgs5*, *Slc2a1*, *Flt1*, *Fn1,* and *Ptprb*) (Fig. 3G). Human microvessel-enriched genes included pericyte and endothelial markers (*TAGLN2, RGS5, FLT1, VWF, FN1,* and *EMCN*) (Fig. 3H). Additional microvessel-enriched transcripts include, *ATP10A, ADGRL4*, *ITM2A*, *NET1*, and *PODXL,* consistent with a role for podocalyxin in BBB maintenance as recently demonstrated in mouse^25^. Whole brain-enriched (microvessel-depleted) genes included *RIMS4*, encoding a neuronal synaptic regulator^26^, *CDH18*, which is also highly expressed in neurons^21^, and *SLC12A5* (KCC2)*,* a potassium-chloride transporter involved in synapse inhibition^27^. Thus, while the discrete set of neural genes examined in Fig. 1 (*GABBR1*, *TUBB3*, and *AQP4*) were not depleted in LCM microvessels, our global differential expression analysis indicated some depletion of neuronal genes.

Gene ontology (GO) analysis of the 100 highest confidence LCM microvessel-enriched genes in mouse and human samples yielded GO terms such as tube morphogenesis, blood vessel morphogenesis/development, and vasculature development, consistent with a vascular transcriptomic signature (Fig. 3I,J). We additionally performed Gene Set Enrichment Analysis (GSEA) on the complete list of human genes ranked from the highest confidence microvessel-enriched gene to the highest confidence microvessel-depleted gene (Supplementary Table S4). Testing this list against 154 gene sets from the KEGG database revealed 13 gene sets enriched in LCM microvessel samples (*P* < 0.05), including the TGFβ signaling pathway, consistent with known roles for TGFβ signaling in vascular biology, BBB development, and endothelial-pericyte interactions^28,29^ (Supplementary Fig. S2). We additionally tested our ranked list against 35 gene sets designed to infer which cell types are present in brain transcriptomic datasets^30^. This analysis confirmed endothelial and mural cell enrichment in microvessel samples, and neuron and astrocyte enrichment in whole brain (Supplementary Fig. S2).

### Mouse-human species differences in vasculature-associated gene expression

**Figure 4:**
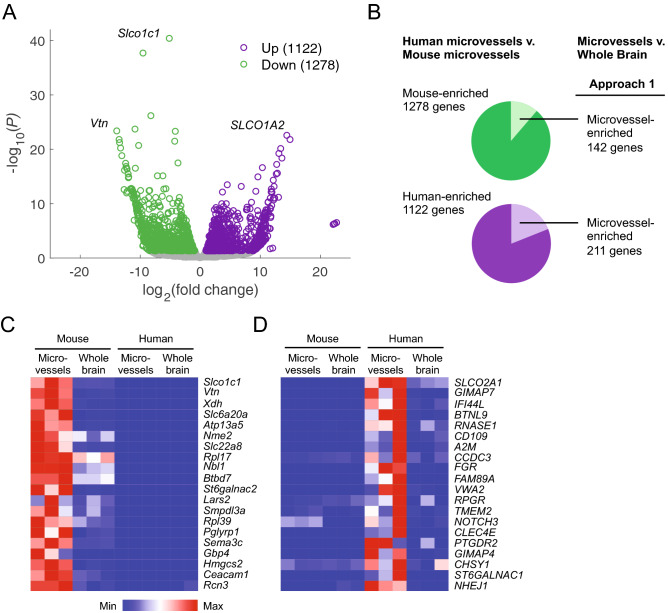
Mouse-human species differences in vasculature-associated gene expression. (**A**) Volcano plot illustrating genes differentially expressed between human LCM microvessels and mouse LCM microvessels. The number of human-enriched (Up) and depleted (Down) genes with adjusted P-values < 0.05 (from DESeq2) are shown in the legend. The complete list of mouse-human gene homology as used in this analysis, and full results of differential expression analysis, are in Supplementary Table S5. (**B**) Summary of filtering strategy used to identify human-mouse species differences potentially attributable to vascular gene expression. Of the 1,278 mouse-enriched transcripts identified in (**A**), Approach 1 selects the 142 genes also enriched in mouse microvessels versus whole brain (as determined in Fig. 3E). Of the 1,122 human-enriched transcripts identified in (**A**), Approach 1 selects the 211 genes also enriched in human microvessels versus whole brain (as determined in Fig. 3F). Complete filtered gene lists are in Supplementary Table S5. Alternative filtering strategies are shown in Supplementary Figure S5. (**C**,**D**) Heat maps illustrating transcript abundance in biological triplicates of mouse and human LCM microvessels and whole brain for the 20 highest confidence mouse-enriched (**C**) and human-enriched (**D**) microvessel genes from lists filtered by Approach 1 (genes also microvessel-enriched in the respective species). Color indicates expression that has been normalized within each gene (row).

Because our mouse and human datasets are derived from samples isolated using identical methods for isolation, sequencing, and analysis, they provide a powerful opportunity to detect species differences in brain microvessel gene expression. We performed a transcriptome-wide comparison of homologous gene expression in the human and mouse LCM microvessel datasets, which revealed 1,122 human-enriched and 1,278 mouse-enriched genes (Fig. 4A; Supplementary Table S5). To identify genes for which microvessel, rather than parenchymal, expression drives species-specific enrichment, we filtered human- and mouse-enriched genes based on microvessel-enrichment in the respective species (Approach 1; Fig. 4B). Of the 1,278 mouse-enriched genes, 142 genes were also microvessel-enriched over mouse whole brain samples, including *Slco1c1* and *Vtn* (Fig. 4B,C; Supplementary Table S5). Notably, expression of *VTN*/*Vtn*, which encodes the extracellular matrix protein vitronectin and has been identified as a pericyte marker in mouse^10,11^, was highly enriched in mouse microvessels (Fig. 4A,C), suggesting human brain pericytes express less *VTN* transcript than mouse pericytes. In fact, we detected no *VTN* transcripts in human microvessels (Supplementary Fig. S3). We confirmed the ability of our pipeline to detect *VTN* transcripts by mapping a human liver RNA-seq dataset^31^ (Supplementary Table S3), which had robust *VTN* expression at 1,330 TPM. We also reviewed two independent human single cell RNA-seq datasets^18,19^, which confirmed a lack of *VTN* expression in human brain pericytes, although these datasets differ in developmental stage and brain region (Supplementary Fig. S3). Similarly, of the 1,122 human-enriched genes, 211 genes were also microvessel-enriched over human whole brain samples, including *SLCO2A1*, *GIMAP7*, *A2M*, *CD109*, *FGR*, *VWA2*, *RNASE1*, and *NHEJ1*. (Fig. 4B,D; Supplementary Table S5). We validated a subset of these human-enriched vascular genes using single cell RNA-seq data from the literature^9,10,18,19^ and immunohistochemistry from the Human Protein Atlas^32^ (Supplementary Fig. S4). For example, the GTPase-encoding transcript *GIMAP7* is not expressed by mouse brain pericytes or endothelial cells, but is expressed by endothelial cells in human embryonic midbrain and neocortex single cell RNA-seq data^18,19^, and Human Protein Atlas data supports vascular localization of the protein in adult human cortex^32^ (Supplementary Fig. S4). Similarly, *A2M*, encoding ⍺-2-macroglobulin, is not expressed by mouse brain pericytes or endothelial cells, but single cell RNA-seq and immunohistochemistry data support its expression in human brain pericytes, endothelial cells, and microglia (Supplementary Fig. S4).

Because Approach 1 eliminates species-specific genes of potential interest which are expressed both in vasculature and parenchyma, we also filtered human- and mouse-enriched gene lists based on the less stringent criterion of no microvessel- or whole brain-enrichment in the respective species (Approach 2; Supplementary Fig. S5; Supplementary Table S5). Further, because Approaches 1 and 2 do not consider genes without known mouse-human homology, or genes with similar expression in microvessels in both species but microvessel-enrichment in only one species, we also identified potential species differences by directly comparing the lists of mouse microvessel-enriched genes (Fig. 3E) and human microvessel-enriched genes (Fig. 3F) (Approach 3; Supplementary Fig. S5; Supplementary Table S5).

This transcriptome-wide analysis identified several solute carrier (*SLC*) transcripts with high differential expression and confidence: *Slco1c1* (Oatp1c1) was highly enriched in mouse microvessels (Fig. 4A,C). Expression of this thyroid hormone transporter in mouse, but not human, brain endothelial cells is consistent with previous observations and explains the lack of central nervous system (CNS) hypothyroidism in mouse models of Allan-Herndon-Dudley Syndrome (AHDS) generated by *Slc16a2* (MCT8) knockout^6^. The amino acid transporter *Slc6a20* and organic anion transporter *Slc22a8* were also highly enriched in mouse microvessels (Fig. 4C). *SLCO1A2* (homologous to mouse *Slco1a5*) was expressed in human microvessels and absent in mouse (Fig. 4A,D), consistent with previous observations of *SLCO1A2* expression in human brain microvessels^33^ and endothelial cells^21^. Given these discrete validated examples and the importance of *SLC* and ATP-binding cassette (*ABC*) transporters in CNS drug delivery, we compared expression of all *SLC* and *ABC* transcripts (Supplementary Fig. S6; Supplementary Table S5). We also found that the ratio of *ABCB1* (mouse *Abcb1a*) to *ABCG2* transcripts, which encode the efflux transporters P-glycoprotein (P-gp) and breast cancer resistance protein (BCRP), respectively, was higher in mouse microvessels (Supplementary Fig. S6), consistent with previous reports^14^. Together, this analysis reveals widespread species differences in microvessel-associated gene expression and demonstrates the utility of whole brain datasets in identifying which species differences are attributable to microvessel gene expression.

### A putative gene expression profile of human brain pericytes

**Figure 5:**
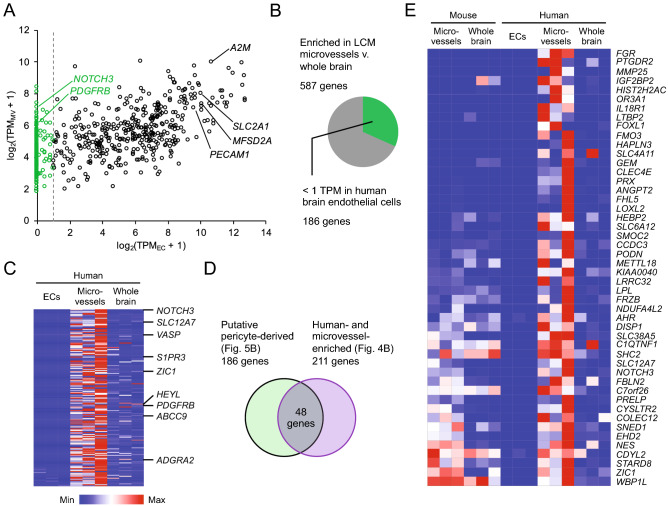
Comparison of human LCM microvessel and brain endothelial cell transcriptomes. (**A**) Average transcript abundance of the 587 human microvessel-enriched genes (as determined in Fig. 3F) in human LCM microvessels (TPM_MV_) versus reference human adult brain endothelial cells analyzed by single cell RNA-seq (TPM_EC_)^17^. Vertical line at log_2_(TPM_EC_ + 1) = 1 indicates the 1 TPM threshold employed to identify putative pericyte-derived transcripts in the microvessel samples. Endothelial genes (e.g. *PECAM1*, *MFSD2A*, *SLC2A1*, *A2M*) fall to the right of this line and are excluded, while known pericyte genes (e.g. *NOTCH3*, *PDGFRB*) fall to the left. Full results of this analysis are in Supplementary Table S6. (**B**) Summary of results of thresholding analysis described in (**A**). (**C**) Heat map illustrating transcript abundance in biological triplicates of human brain endothelial cells and LCM microvessels and whole brain for the 186 putative pericyte genes identified in (**A**). Known and putative novel pericyte genes are annotated. Color indicates expression that has been normalized within each gene (row). (**D**) Summary of filtering strategy used to identify putative human-specific pericyte genes. Of the 186 putative pericyte genes identified in (**A**), 48 are also human-enriched (as determined in Fig. 4B). (**E**) Heat map illustrating transcript abundance in biological triplicates of mouse LCM microvessels and whole brain, human brain endothelial cells, and human LCM microvessels and whole brain for the 48 putative human-enriched pericyte genes identified in (**D**). Genes are ranked by fold change in human versus mouse microvessels. Color indicates expression that has been normalized within each gene (row).

Pericytes play important roles in brain vascular function and BBB development^34–36^. While recent work has characterized the transcriptional profiles of mouse brain pericytes^10^, human brain pericytes after in vitro culture^37^, and small numbers of human embryonic brain pericytes^18,19^, a comprehensive molecular definition of adult human brain pericytes in vivo is lacking. We reasoned that because our human LCM microvessel samples contain both endothelial and pericyte-derived transcripts, we could identify genes putatively expressed by human brain pericytes by subtractive comparison of our microvessel datasets to existing transcriptomic data from purified human brain endothelial cells (Supplementary Table S3)^17^. Similar strategies have been used to identify mouse brain pericyte enriched-genes from microvessel and pericyte-depleted microvessel transcriptome datasets^8,38^. We compared the expression of 587 human LCM microvessel-enriched genes identified in Fig. 3F to an adult human temporal lobe brain endothelial single cell RNA-seq dataset^17^ (Fig. 5A). Human LCM microvessel-enriched genes that had an expression level of 1 TPM or greater in the reference endothelial cell dataset were considered endothelial-derived and excluded. The remaining LCM microvessel-enriched genes were then categorized as putative pericyte genes. This analysis resulted in 186 putative pericyte genes (Fig. 5B), including known pericyte markers (*PDGFRB*, *NOTCH3*, *S1PR3*, *ABCC9*, *ADGRA2* [GPR124]) and excluding known endothelial genes (*PECAM1*, *A2M*, *SLC2A1*, *MSFD2A*) (Fig. 5A,C; Supplementary Table S6).

To identify potential species differences in pericyte gene expression, we compared this putative pericyte-enriched gene list to the human-specific microvessel-enriched gene list (Fig. 4B) and found 48 such human-enriched putative pericyte genes (Fig. 5D,E). We evaluated expression of a subset of these putative human pericyte genes using immunohistochemical analysis from the Human Protein Atlas^32^ and several independent RNA-seq datasets from the literature: mouse brain vascular single cell RNA-seq^9,10^, human embryonic midbrain single cell RNA-seq^18^, human embryonic neocortex single cell RNA-seq^19^, and adult human cortex single nucleus RNA-seq^39^ (Supplementary Fig. S7). These data support expression of *SLC6A12*, *SLC12A7*, *GEM*, *FRZB*, *FOXL1*, *PTGDR2*, *FHL5*, *SMOC2*, and *LPL* in human brain pericytes in at least one validation dataset, and suggest lower or minimal expression in mouse brain pericytes. For example, the human single cell RNA-seq and immunohistochemistry datasets corroborated our findings indicating pericyte expression of *SLC12A7* in human cortex and midbrain (Supplementary Fig. S7). Notably, mouse single cell RNA-seq data suggests that *Slc12a7* is expressed by a small number of pericytes and endothelial cells^10^, in contrast to humans where virtually no endothelial expression was detected (Supplementary Fig. S7). Similarly, *GEM* (encoding a GTP-binding protein), *FRZB* (encoding a Wnt-binding protein) and the transcription factor *FOXL1* were absent or nearly so in mouse brain pericytes but robustly expressed by human brain pericytes (Supplementary Fig. S7). Other genes (*MMP25*, *ANGPT2*) were expressed in both human endothelial cells and pericytes but nearly absent in the corresponding mouse cell types (Supplementary Fig. S7).

This multi-dataset validation also revealed that some genes, including *ZIC1*, *NOTCH3*, *PRELP*, *NDUFA4L2*, *LRRC32*, *COLEC12*, *KIAA0040,* and *C1QTNF1* are expressed by both mouse and human pericytes; thus, our analysis suggests that these genes may be enriched in relative abundance in human microvessels versus mouse (Supplementary Fig. S7). Additionally, *EHD2*, while expressed at similar levels by mouse brain endothelial cells and pericytes, was highly pericyte-enriched in human single cell/nucleus RNA-seq datasets (Supplementary Fig. S7). *PRX* (periaxin), a gene required for myelination by Schwann cells^40^, was recently identified as expressed in human brain microvasculature by immunohistochemistry^41^, but *Prx* transcripts were nearly absent in mouse microvessels and mouse brain pericytes and endothelial cells (Supplementary Fig. S7). We again used independent, human single cell RNA-seq datasets to corroborate these findings, and found very low *PRX* expression in both endothelial cells and pericytes despite strong vascular localization in the immunohistochemistry dataset (Supplementary Fig. S7). Finally, some genes (*HAPLN3*, *SLC38A5*, *STARD8*) were indeed expressed by endothelial cells in these validation datasets, suggesting an erroneous lack of expression in the initial endothelial reference dataset or potentially reflecting differences in developmental stage or brain region (Supplementary Fig. S7), highlighting the importance of evaluating multiple datasets.

Together, this analysis demonstrates the utility of human LCM microvessel and whole brain datasets in identifying potential human pericyte-derived transcripts, suggests additional mouse-human species differences in endothelial and pericyte gene expression, and motivates the integration of several independent RNA-seq datasets to build confidence in cell type-specific gene expression. Further work will be required to confirm expression, cellular origin, and potential functional roles of genes identified in this analysis in the human adult brain vasculature.

## Discussion

In this study, we isolated human and mouse brain microvessels and used RNA-seq to characterize the transcriptomes of these samples. LCM yielded microvessel samples containing the two cell types sharing the microvascular basement membrane, BMECs and pericytes. We observed some expression of neuronal and glial marker genes in LCM microvessel datasets, consistent with the technically unavoidable capture of some parenchymal cells. The common issue of multiple or contaminating cell types in tissue-derived RNA-seq samples has been largely obviated by single cell RNA-seq; however, single cell RNA-seq presents other challenges such as poor ability to detect very low abundance transcripts and potential transcriptomic alterations induced by cell dissociation and purification^42^. Thus, bulk and single cell RNA-seq are complementary strategies, and LCM has been used previously to isolate human blood-nerve barrier endoneurial microvessels from patient samples for downstream transcriptomic profiling^43^. To further validate our mouse LCM microvessel datasets, we used data from recent single cell RNA-seq of mouse brain endothelial cells and pericytes^9,10^. We found the highest correlation between our data at an approximately 1:1 combination of endothelial cell- and pericyte-derived transcripts, approximately consistent with the ratio of the two cell types in mouse brain microvasculature^44^, assuming similar global transcript abundance in BMECs and pericytes. The maximum transcriptome-wide correlation we observed, however, had a Pearson correlation coefficient of only 0.34, consistent with the presence of parenchyma-derived transcripts in our LCM microvessel samples. Thus, the RNA-seq of matched whole brain samples is paramount in identifying endothelial and pericyte genes based on microvessel-enriched expression. Furthermore, while numerical methods to estimate the relative population of different cell types from such datasets are available^45,46^, they require reliable reference datasets for the cell types present. The extremely limited number and depth of human brain endothelial and pericyte RNA-seq datasets currently precludes the application of such techniques to our human LCM microvessel data.

Further analysis of our datasets via PCA and hierarchical clustering demonstrated expected similarity between three biological replicates of mouse microvessels, which clustered distinctly from whole brain. Similar analyses of the human datasets produced no such predictable clustering, demonstrating patient-to-patient variability, differences in sampling location, or different effects of neurological condition. Despite this, results of transcriptome-wide differential expression analysis, GO analysis, and GSEA support depletion of neural-associated genes and enrichment of vasculature-associated genes in both human and mouse LCM microvessel datasets compared to whole brain in a pairwise differential expression analysis. Overall, though there was high variability between human samples, LCM microvessel datasets were enriched in known BMEC and pericyte genes, confirming the utility of both the isolation method and the resulting datasets. The observed variability in human samples and uncertainty of sample location motivates future efforts to characterize additional patient samples. Additional matched samples would be required to power a study to understand how brain vascular gene expression varies with factors such as brain region, age, or disease, factors that have only recently begun to be explored in mouse^10,38^.

To demonstrate the utility of the resulting datasets, we identified species-specific differences in brain vascular gene expression. Species differences in neural progenitor, neuronal, and astrocyte gene expression have been characterized by RNA-seq^18,21^, but such differences in vascular gene expression have not been comprehensively analyzed. In addition to a transcriptome-wide comparison of homologous gene expression, we used the paired whole brain datasets to identify only those genes with species-specific expression attributable to the vasculature and not the brain parenchyma. In this way, we identified 142 mouse-enriched genes, including *Vtn*, *Slco1c1*, *Slc6a20a*, *Atp13a5*, *Slc22a8*, and 211 human-enriched genes, including *SLCO2A1*, *GIMAP7*, and *A2M*. Given that BBB efflux by *ABC* transporters hinders delivery of many small molecule drugs, and *SLC* transporters may facilitate transport of some pharmaceuticals, it is important to understand differences in expression between human and mouse for drug development^14,15,20,47^. Additionally, recent work demonstrated an enrichment of transporter genes in mouse brain pericytes compared to mouse lung pericytes, suggesting that brain pericytes may be directly involved in molecular transport^10^. Thus, our data permit identification of putative human pericyte-enriched transporters that may be involved in molecular transport across the human BBB, such as the potassium-chloride cotransporter *SLC12A7* and the betaine transporter *SLC6A12* identified in our analysis. Further, improved insight into differences in gene expression between mouse and human brain vasculature is key to assess potential limitations of mouse models of brain vascular and BBB development and disease, as is the case in AHDS^6^. For example, we found that *VTN*, a gene highly and selectively expressed in mouse brain pericytes with a recently-described functional role in neurogenesis^48^, was not expressed at a detectable level by human brain pericytes. These results were validated in transcriptomics data collected by other groups, though these datasets vary in developmental stage and brain region^18,19^. We also implemented two alternative approaches to identify putative species differences (Supplementary Fig. S5), but note that because of variability between human samples, our analyses likely identified only the most robustly differentially-expressed genes, and that future studies with increased sample size could illuminate additional mouse-human species differences in vascular gene expression. Taken together, availability of human vascular transcriptomes can aid investigators in identifying potential roles for BMECs and pericytes in the etiology of neurological disorders with known genetic bases and should allow others to quickly assess expression of molecules with potential relevance to drug delivery (e.g. *SLC*s, *ABC*s, and large molecule receptors) and disease in human brain vasculature.

Finally, we demonstrated how human brain microvessel transcriptome datasets could be further mined for pericyte-expressed genes by comparison to existing brain endothelial transcriptomes^17^. We identified several genes enriched in our human LCM microvessel datasets compared to both whole brain and existing human brain endothelial cell datasets, such as *SLC6A12*, *SLC12A7*, *PRELP*, *NDUFA4L2*, *GEM*, *FRZB*, *LRRC32*, *EHD2*, *FOXL1*, *COLEC12*, *KIAA0040*, *PTGDR2*, *C1QTNF1*, *FHL5*, *SMOC2*, and *LPL*, suggesting these as possible human pericyte-expressed genes, some of which are human-specific. For example, *SLC12A7*, which is weakly expressed in both pericytes and endothelial cells in mouse brain vasculature, was strongly and selectively expressed by human brain pericytes in single-cell RNA-seq datasets, and had vascular localization in immunohistochemistry data from the Human Protein Atlas. These results motivate the use of several independent datasets to account for patient-to-patient variability and technical differences in cell isolation and sequencing methodologies to validate putative human pericyte and BMEC gene expression. Our human LCM microvessel datasets should facilitate hypothesis generation and future validation of hypothesized human pericyte-expressed transcripts with potential roles in vascular function or disease.

Overall, this work contributes brain microvessel transcriptome datasets enriched in BMEC and pericyte genes from three human patient and three mouse samples. We also contribute matched whole brain datasets to aid in identification and assessment of microvessel-associated genes. Further, our work demonstrates the utility of LCM as a valuable tool for isolation and molecular analysis of brain microvasculature. Finally, we have demonstrated application of our datasets to identify differences between mouse and human brain vasculature with potential implications for drug delivery and disease, and to discover previously unknown human BMEC- and brain pericyte-expressed genes within these relatively unexplored transcriptomes.

## Methods

### Human tissue

This analysis was performed on de-identified, normal human brain tissue that is usually discarded during surgery to access deeper diseased brain regions. Human brain tissue was obtained during three surgeries for other indications under a University of Wisconsin-Madison Institutional Review Board-approved protocol. All research was conducted in accordance with the guidelines and supervision of the University of Wisconsin-Madison’s Institutional Review Board. The obtained tissue specimens were flash-frozen in liquid nitrogen and stored at −80 °C until sectioned.

### Mouse tissue

All procedures were performed in compliance with the University of Wisconsin-Madison Animal Care and Use Committee and following National Institutes of Health (NIH) guidelines for care and use of laboratory animals. Mouse brain tissue was obtained from C57/BL6N mice aged 6–7 weeks, and tissue was flash-frozen in liquid nitrogen and stored at −80 °C until sectioned.

### Laser capture microdissection and RNA extraction

Tissues were sectioned onto RNase-free membrane slides (Molecular Machines & Industries) using RNase-free techniques at a thickness of 8 μm and stored at −80 °C until proceeding to laser capture. Tissue sections were used within 1 week of sectioning to prevent RNA degradation.

All subsequent procedures were performed at room temperature using RNase-free techniques. Slides were thawed for 1.5 min, fixed in 100% acetone (Sigma) for 2 min, and air dried for 2 min. Capillaries were labeled for 2 min using a lectin stain: mouse tissue was stained with fluorescein labeled *Rincinus communis* Agglutinin I (RCA-I, 1:20, Vector Labs), and human tissue was stained with fluorescein labeled *Ulex europaeus* Agglutinin I (UEA-I, 1:10, Vector Labs) diluted in water. Slides were washed 3 times with water and placed in a desiccator for 10 min. Slides were then dehydrated via two 30 s incubations in 95% ethanol (Sigma) followed by two 30 s incubations in 100% ethanol and finally a 3 min incubation in 100% isopropyl alcohol (Sigma). Slides were placed in a desiccator for another 3 min before proceeding to capture. The lectin-labeled blood vessels were cut from the sections and captured on an adhesive cap (Molecular Machines and Industries) using the MMI Cell Cut instrument (Molecular Machines and Industries). Only microvessels (≤ 10 μm in diameter) were selected for capture.

A total of 2.2–3.0 mm^2^ of mouse brain microvessels and 2.3–3.0 mm^2^ of human brain microvessels were collected per biological replicate, and three biological replicates were obtained. An equivalent area of whole brain tissue was captured onto an adhesive cap from each sample as a control. Captured tissue was lysed with buffer RLT (Qiagen) supplemented with β-mercaptoethanol (Sigma), vortexed for 30 s, and stored at –80 °C until RNA extraction.

For RNA isolation, lysed tissue was thawed at room temperature and total RNA was extracted using an RNeasy Micro kit (Qiagen) per the manufacturer’s instructions. On-column DNase I digestion was performed to remove genomic DNA. RNA was quantified on an Agilent Bioanalyzer using the RNA 6,000 Pico kit, and electropherogram presence of 18S and 28S ribosomal RNA was used to confirm intact RNA prior to library construction and sequencing (Supplementary Fig. S1).

### RNA-sequencing and data analysis

Sequencing libraries enriched for non-rRNA sequences were constructed using the NuGEN Ovation Single Cell RNA-Seq High Volume Beta Kit. Libraries were sequenced on an Illumina HiSeq 2000 sequencer using 100 bp single-end reads. Between 19 and 33 million reads were collected per sample (Supplementary Table S1).

Raw FASTQ files from our sequencing or obtained from NCBI SRA (for reference dataset accession numbers, see Supplementary Table S3) were mapped to the mouse genome (mm10) or human genome (hg38) using STAR^49^ (version 2.6.0b-1) implemented in Galaxy^50^ on the public server at https://usegalaxy.org. Gene-level transcript abundances were calculated using featureCounts^51^ (version 1.6.3) also implemented in Galaxy.

Differential expression analysis was performed using DESeq2^52^ (version 3.10), implemented in R (version 3.6.2), using raw counts as input. When comparing LCM microvessel and whole brain samples, sample pairing was included in the DESeq2 design. Elsewhere in the paper, transcript abundances are presented as transcripts per million (TPM). For each gene *i*, TPM_*i*_ was calculated as

1 $$ TPM_{i} = \frac{{FPKM_{i} }}{{\sum\nolimits_{i} {FPKM_{i} } }} \times 10^{6} $$

where fragments per kilobase of transcript per million mapped reads (FPKM) was calculated as

2 $$ FPKM_{i} = \frac{{{\text{counts}}_{i} }}{{L_{i} \sum\nolimits_{i} {{\text{counts}}_{i} } }} \times 10^{6} $$

where *L*_*i*_ is the length of the transcript, in kilobases, reported by featureCounts, and sums run over all genes in a single sample. Count, FPKM, and TPM data for our datasets and reference datasets are provided in Supplementary Table S2. All P-values presented in the context of differential expression analyses are those adjusted for multiple comparisons by DESeq2 using the Benjamini–Hochberg procedure.

Hierarchical clustering on TPM was performed with GENE-E (version 3.0.215), using the one minus Pearson correlation with average linkage. Principal component analysis on TPM was performed using MATLAB. GO analysis was performed using the PANTHER^53^ online tool at https://pantherdb.org. Gene Set Enrichment Analysis (GSEA)^54,55^ was performed using a list of genes ranked based on DESeq2 output, using the ranking metric − log_10_(*P*) × sign[log_2_(fold change)], which organizes high-confidence microvessel enriched genes at the top of the list, and high-confidence microvessel-depleted genes at the bottom.

### Human-mouse gene expression comparison

To compare gene expression in human LCM microvessels and mouse LCM microvessels, a human-mouse gene homology database was obtained from Mouse Genome Informatics (https://www.informatics.jax.org/homology.shtml) and manually curated to match gene symbols to those in featureCounts outputs derived from genome annotation files. Human genes with no mouse homolog and mouse genes with no human homolog were not considered in this analysis. The majority (98%) of genes had 1:1 mouse-human homology. For human genes with multiple mouse homologs, TPM values for the mouse homologs were summed. For mouse genes with multiple human homologs, either (i) the single human homolog with the highest TPM was retained and the human homolog(s) with zero or negligible expression were eliminated, or (ii) if multiple human homologs existed with similar expression, these were each compared individually to the mouse homolog. Supplementary Table S5 contains a complete list of mouse-human homology as used in this analysis. Because mouse and human homologs for a given gene differ in transcript length, raw counts from each dataset do not permit an authentic comparison of expression. Thus, as a DESeq2 input, we used TPM values to reverse-calculate an unnormalized counts metric for each gene in mouse and human samples using the human gene length. Other comparisons of mouse and human gene expression were performed using TPM values.

### Comparison to single cell RNA-seq reference datasets

Raw counts from mouse brain endothelial single-cell RNA-seq datasets^10^ (Supplementary Table S3) were averaged across all single cells and normalized using transcript lengths to generate a single vector of TPM values. The same process was used for mouse brain pericyte single-cell RNA-seq datasets (Supplementary Table S3). We mathematically generated new vectors of TPM values by combining these reference datasets in different pericyte-to-endothelial ratios as

3 $$ TPM_{\alpha } = \alpha \left( {TPM_{{{\text{peri}}}} } \right) + \left( {1 - \alpha } \right)\left( {TPM_{{{\text{endo}}}} } \right) $$

where *α* represents the fraction of transcripts derived from pericytes and varies between 0 and 1. To assess global similarity between reference datasets and LCM microvessel data, Pearson and Spearman’s correlation coefficients were calculated on TPM values using MATLAB.

### Validation of pericyte-enriched transcripts

Human and mouse brain single-cell and single-nucleus RNA-seq datasets^9,10,18,19,39^ were used for validation of putative pericyte-enriched transcripts, with cell assignments to clusters as reported by the authors. Seurat (version 3.1.2)^56^ implemented in R was used for visualization except where indicated in figure legends. Images from the Human Protein Atlas (https://v19.proteinatlas.org)^32^ were also used for validation.

### Statistical analyses

DESeq2 was used for all differential expression analyses as described above, and all P-values reported in the context of differential expression analyses are those generated by DESeq2 using the Wald test and adjusted to control the false discovery rate using the Benjamini–Hochberg procedure, except where otherwise indicated. P-values reported for Pearson correlation coefficients were calculated in MATLAB using Student’s *t* distribution. P-values reported for GO and GSEA analyses were those generated by the tools as described above. Student’s *t* test was used to compare the ratio of *ABCB1* and *ABCG2* transcripts (Supplementary Fig. S6).

## Supplementary information

Supplementary Figures S1 to S7 and Table S1.

Supplementary Table S2.

Supplementary Table S3.

Supplementary Table S4.

Supplementary Table S5.

Supplementary Table S6.

## Data Availability

Sequencing data have been submitted to the Gene Expression Omnibus with accession number GSE142209. All processed data are available in the paper or supplementary information.
