# Supplementary material for: Transcriptomic comparison of human and mouse brain microvessels
Source: Sci Rep. 2020 Jul 23;10:12358. doi: 10.1038/s41598-020-69096-7 (PMC7378255; doi:10.1038/s41598-020-69096-7)
Supplement: Supplementary file 1 — Supplementary Figures S1 to S7 and Table S1. [file 41598_2020_69096_MOESM1_ESM.pdf]

Transcriptomic Comparison of Human and Mouse Brain Microvessels

Hannah W. Song, Koji L. Foreman, Benjamin D. Gastfriend, John S. Kuo, Sean P. Palecek and Eric V. Shusta

Supplementary information.

- **Figure S1.** RNA electropherograms.
- **Figure S2.** Gene Set Enrichment Analysis (GSEA) of human LCM microvessel datasets.
- **Figure S3.** Lack of *VTN* expression in human brain pericytes.
- **Figure S4.** Validation of putative human-enriched vascular transcripts.
- **Figure S5.** Alternative filtering strategies for identifying species differences in vascular gene expression.
- **Figure S6.** Mouse-human species differences in *SLC* and *ABC* transporter expression.
- **Figure S7.** Validation of putative human brain pericyte-enriched transcripts.
- **Table S1.** Characteristics of RNA samples and associated RNA-seq datasets.

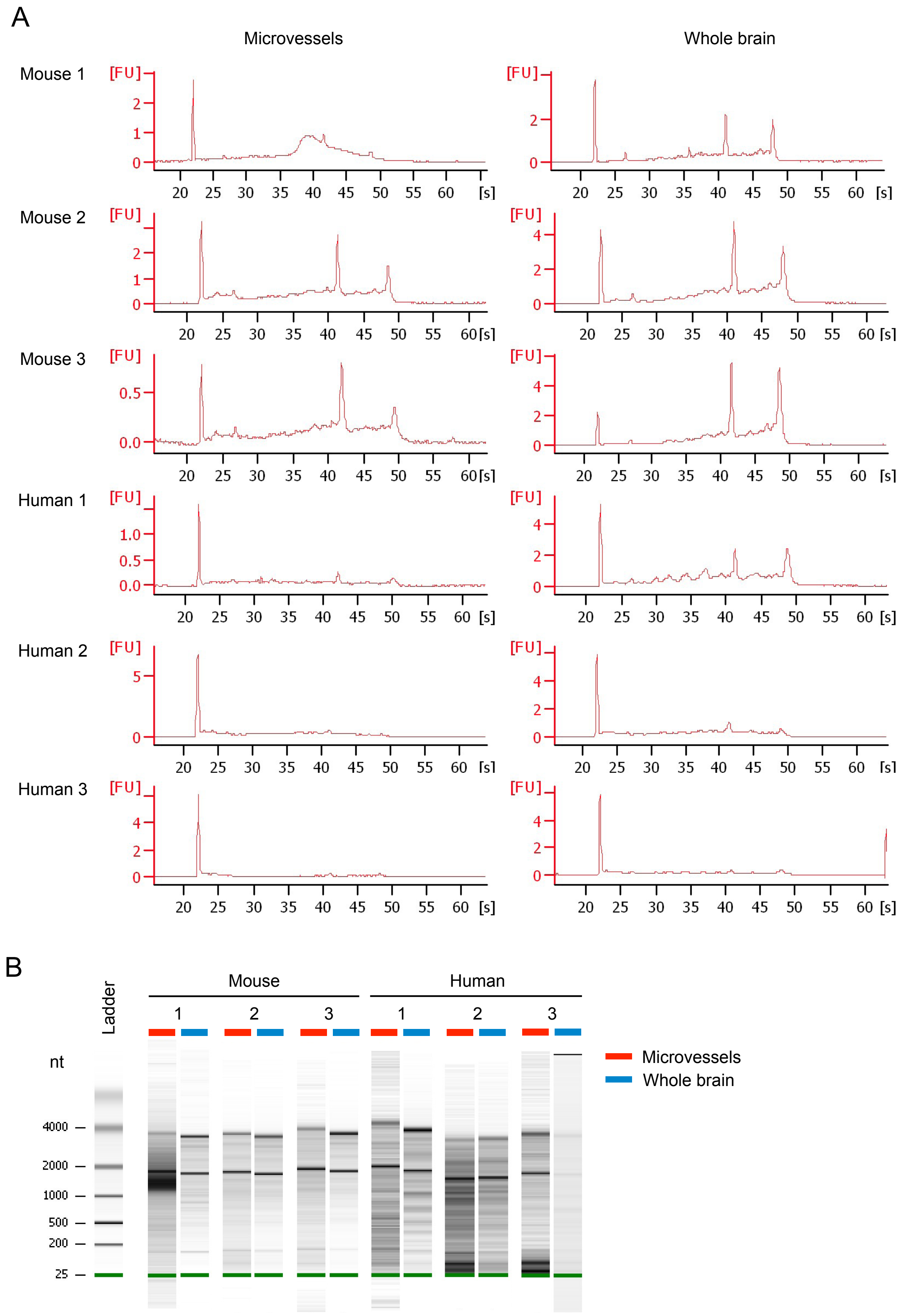
A
Microvessels
Whole brain
Mouse 1
Mouse 2
Mouse 3
Human 1
Human 2
Human 3
[FU]
[s]
B
Ladder
Mouse
Human
1
2
3
nt
4000
2000
1000
500
200
25
Microvessels
Whole brain

Figure S1. RNA electropherograms. (A,B) Agilent Bioanalyzer analysis of RNA samples from mouse and human LCM microvessel and whole brain samples. Electropherograms (A) and gel-like images (B) are shown.

A

Gene sets (KEGG) Microvessel-enriched	NES	*P*	FDR
TGF-beta signaling pathway	1.74	0	0.0040
Focal adhesion	1.74	0	0.0034
Dorso-ventral axis formation	1.63	0	0.062
Cytokine-cytokine receptor interaction	1.62	0	0.065
Complement and coagulation cascades	1.60	0.016	0.075
Selenoamino acid metabolism	1.58	0.011	0.076
Adipocytokine signaling pathway	1.53	0	0.14
Arachidonic acid metabolism	1.50	0.030	0.18
Vascular smooth muscle contraction	1.47	0.022	0.25
ECM-receptor interaction	1.44	0.018	0.30
Arrhythmogenic right ventricular cardiomyopathy	1.40	0.046	0.37
Leukocyte transendothelial migration	1.36	0.046	0.41
Small cell lung cancer	1.35	0.050	0.43

B

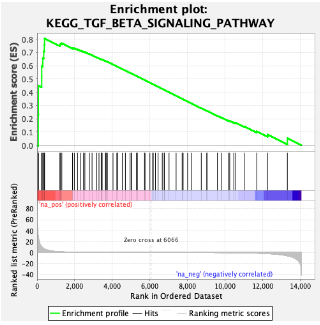

C

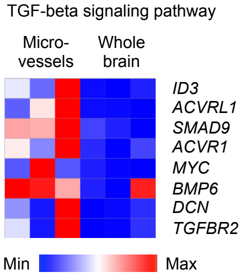

D

Gene sets (Hagenauer *et al.* 2018) Microvessel-enriched	NES	*P*	FDR
Endothelial all, Zeisel Science 2015	2.18	0	0
Endothelial all, Daneman PLoS 2010	1.75	0	0.00069
Endothelial all, Zhang JNeuro 2014	1.75	0	0.00046
Endothelial all, Darmanis PNAS 2015	1.69	0	0.0019
Neuron corticospinal, Doyle Cell 2008	1.59	0.0088	0.0094
Mural pericyte, Zhang JNeuro 2014	1.49	0.053	0.046

Gene sets (Hagenauer *et al.* 2018) Microvessel-depleted	NES	*P*	FDR
Neuron interneuron, Zeisel Science 2015	-1.71	0	0
Neuron all, Darmanis PNAS 2015	-1.45	0.011	0.059
Astrocyte all, Zhang JNeuro 2014	-1.39	0.028	0.12
Neuron all, Cahoy JNeuro 2008	-1.36	0.048	0.15

E

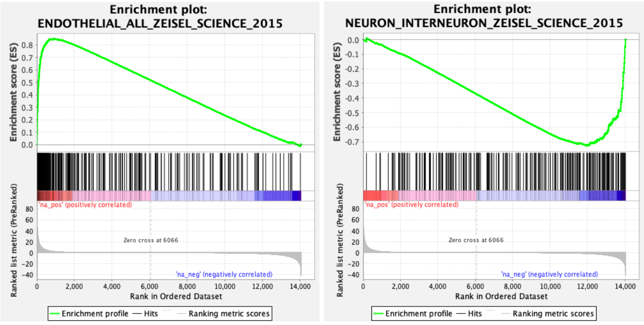

F

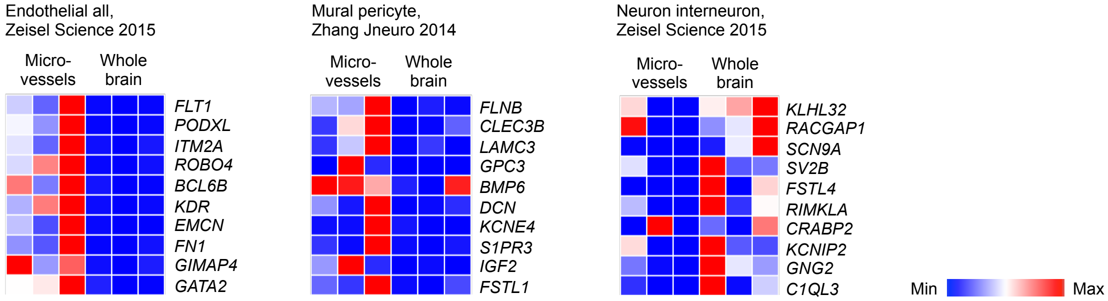

Figure S2. Gene Set Enrichment Analysis (GSEA) of human LCM microvessel datasets. (A) Gene sets from the KEGG database enriched (*P* or FDR < 0.05) in human LCM microvessels compared to whole brain. NES: normalized enrichment score. The GSEA input was a list of

genes ranked from the highest-confidence LCM microvessel-enriched to the highest-confidence LCM microvessel-depleted gene, using the ranking metric $-\log_{10}(P)\times\text{sign}[\log_2(\text{fold change})]$ (Supplementary Table S4). **(B)** GSEA enrichment plots for the KEGG TGF-beta signaling pathway gene sets. **(C)** Heat maps illustrating transcript abundance in biological triplicates of LCM microvessels and whole brain for the highest ranked LCM microvessel-enriched genes in the indicated gene sets. Color indicates expression that has been normalized within each gene (row). **(D)** Gene sets from Hagenauer *et al.* [30] enriched (P or FDR < 0.05) in human LCM microvessels compared to whole brain (top) and enriched (P or FDR < 0.05) in whole brain compared to LCM microvessels (bottom). GSEA input was as described in (A). **(E)** GSEA enrichment plots for endothelial and interneuron gene sets from Hagenauer *et al.* [30]. **(F)** Heat maps illustrating transcript abundance in biological triplicates of LCM microvessels and whole brain for the highest ranked LCM microvessel-enriched genes in the indicated gene sets. Color indicates expression that has been normalized within each gene (row).

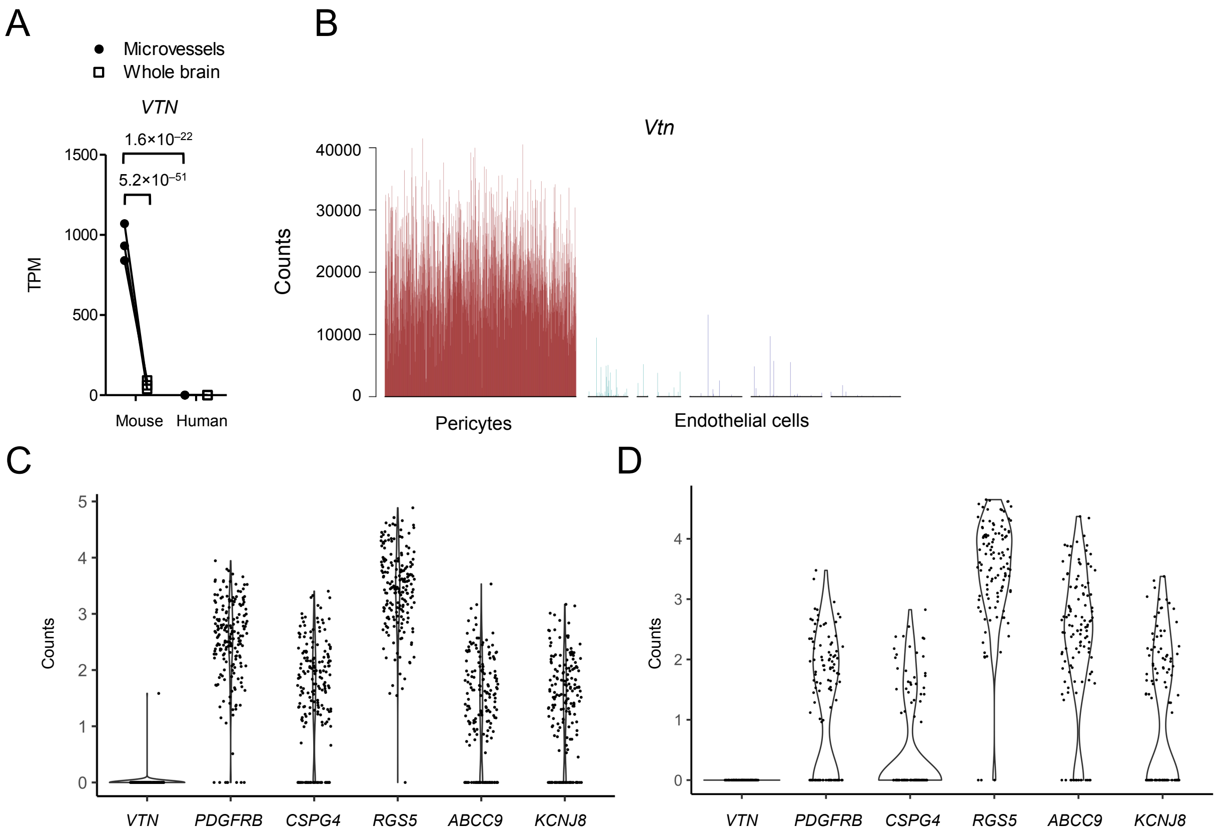

Figure S3. Lack of *VTN* expression in human brain pericytes. (A) Transcript abundance of *VTN/Vtn* in biological triplicates of LCM microvessels and whole brain from human and mouse. Lines connect datapoints from matched LCM microvessel and whole brain samples. Adjusted P-values (from DESeq2) are shown; Wald test with Benjamini-Hochberg correction. **(B)** *Vtn* expression in single cell RNA-seq of mouse brain pericytes and endothelial cells [9,10]. Single cell RNA-seq plot in this panel adapted from http://betsholtzlab.org/VascularSingleCells/database.html. **(C,D)** *VTN* expression in human brain pericytes. Other pericyte genes (*PDGFRB*, *CSPG4*, *RGS5*, *ABCC9*, and *KCNJ8*) are included for comparison. Single cell RNA-seq data from (C) La Manno *et al.* [18] and (D) Polioudakis *et al.* [19].

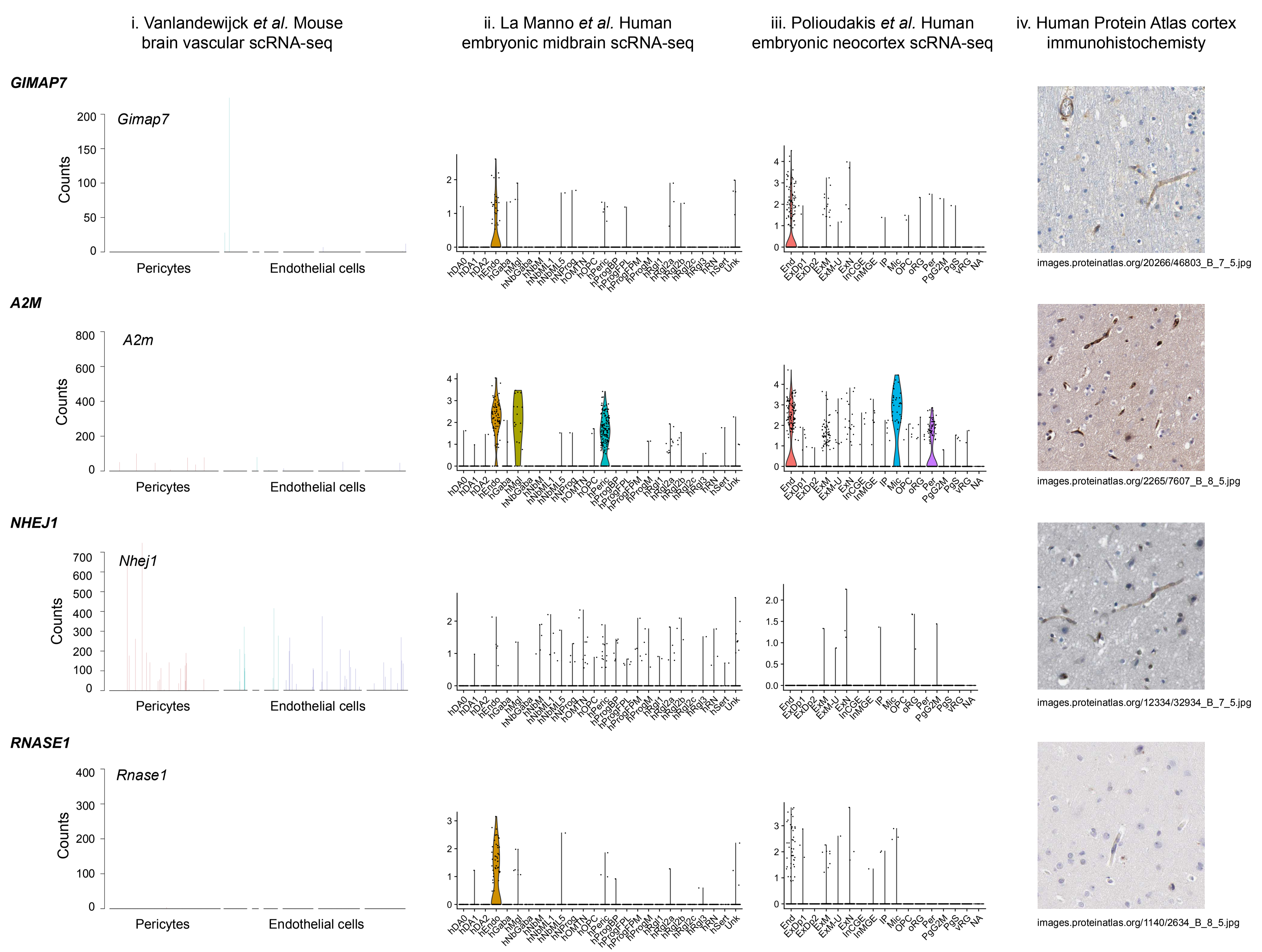

i. Vanlandewijck et al. Mouse brain vascular scRNA-seq
ii. La Manno et al. Human embryonic midbrain scRNA-seq
iii. Polioudakis et al. Human embryonic neocortex scRNA-seq
iv. Human Protein Atlas cortex immunohistochemisty
GIMAP7
Gimap7
Counts
Pericytes
Endothelial cells
images.proteinatlas.org/20266/46803_B_7_5.jpg
A2M
A2m
Counts
Pericytes
Endothelial cells
images.proteinatlas.org/2265/7607_B_8_5.jpg
NHEJ1
Nhej1
Counts
Pericytes
Endothelial cells
images.proteinatlas.org/12334/32934_B_7_5.jpg
RNASE1
Rnase1
Counts
Pericytes
Endothelial cells
images.proteinatlas.org/1140/2634_B_8_5.jpg
hDA0 hDA1 hDA2 hEndo hGaba hMgl hNbGaba hNbM hNbML1 hNbML5 hNProg hOMTN hOPC hPeric hProgBP hProgFPL hProgFPM hProgM hRgl1 hRgl2a hRgl2b hRgl2c hRgl3 hRN hSert Unk
End ExDp1 ExDp2 ExM ExM-U ExN InCGE InMGE IP Mic OPC oRG Per PgG2M PgS vRG NA

Figure S4. Validation of putative human-enriched vascular transcripts. Each row shows data from several sources for one transcript/protein of interest. (i) Transcript expression in single cell RNA-seq of mouse brain pericytes and endothelial cells [9,10]. Single cell RNA-seq plots in these panels adapted from http://betsholtzlab.org/VascularSingleCells/database.html. (ii) Transcript abundance (counts) in human embryonic midbrain single cell RNA-seq data [18]. Cell assignment to clusters and cluster names are as reported by La Manno *et al.* [18]. hPeric: human pericytes. hEndo: human endothelial cells. (iii) Transcript abundance (counts) in human embryonic neocortex single cell RNA-seq data [19]. Cell assignment to clusters and cluster names are as reported by Polioudakis *et al.* [19]. Per: pericytes. End: endothelial cells. (iv) Immunohistochemistry on human cortex samples from the Human Protein Atlas (v19.proteinatlas.org) [32]. URLs for each image are provided in the figure.

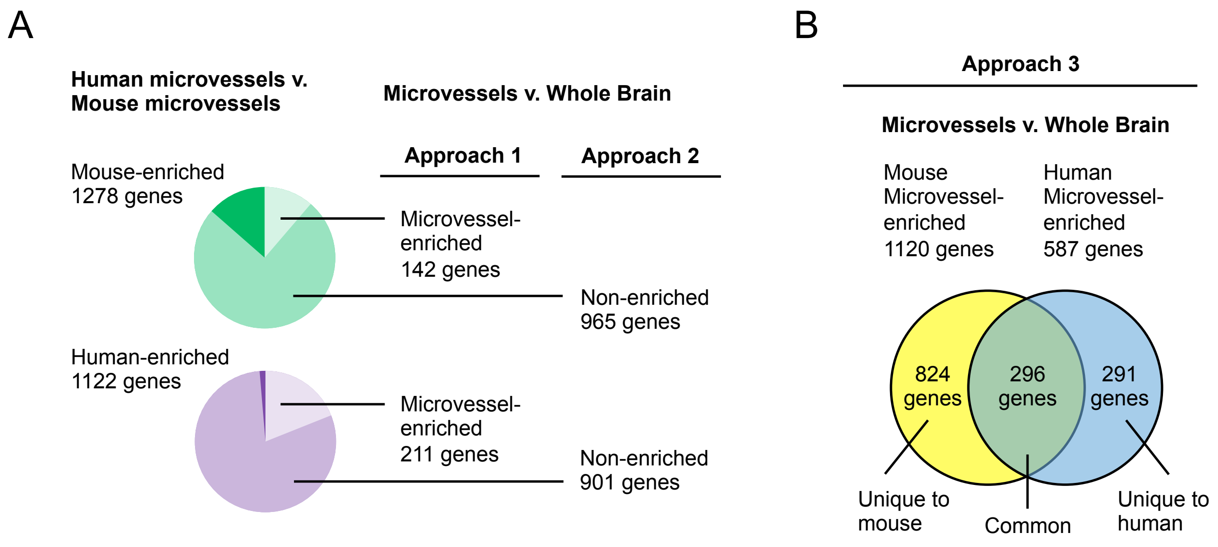

Figure S5. Alternative filtering strategies for identifying species differences in vascular gene expression. (A) Summary of Approaches 1 and 2. Approach 1 is as shown in Figure 4. Of the 1278 mouse-enriched transcripts identified in (A), Approach 2 selects the 965 genes not enriched in mouse microvessels or whole brain (as determined in Fig. 3E). Of the 1122 human-enriched transcripts identified in (A), Approach 2 selects the 901 genes not enriched in human microvessels or whole brain (as determined in Fig. 3F). The small number of genes not selected by Approach 1 or Approach 2 (shaded dark) are whole brain-enriched. Complete filtered gene lists are in Supplementary Table S5. **(B)** Summary of Approach 3, a filtering strategy that identifies species-specific microvessel-enriched genes by comparing the lists of mouse genes with microvessel enrichment versus whole brain (as determined in Fig. 3E) and human genes with microvessel enrichment versus whole brain (as determined in Fig. 3F). 824 genes appear only on the mouse list and 291 genes appear only on the human list. Complete filtered gene lists are in Supplementary Table S5.

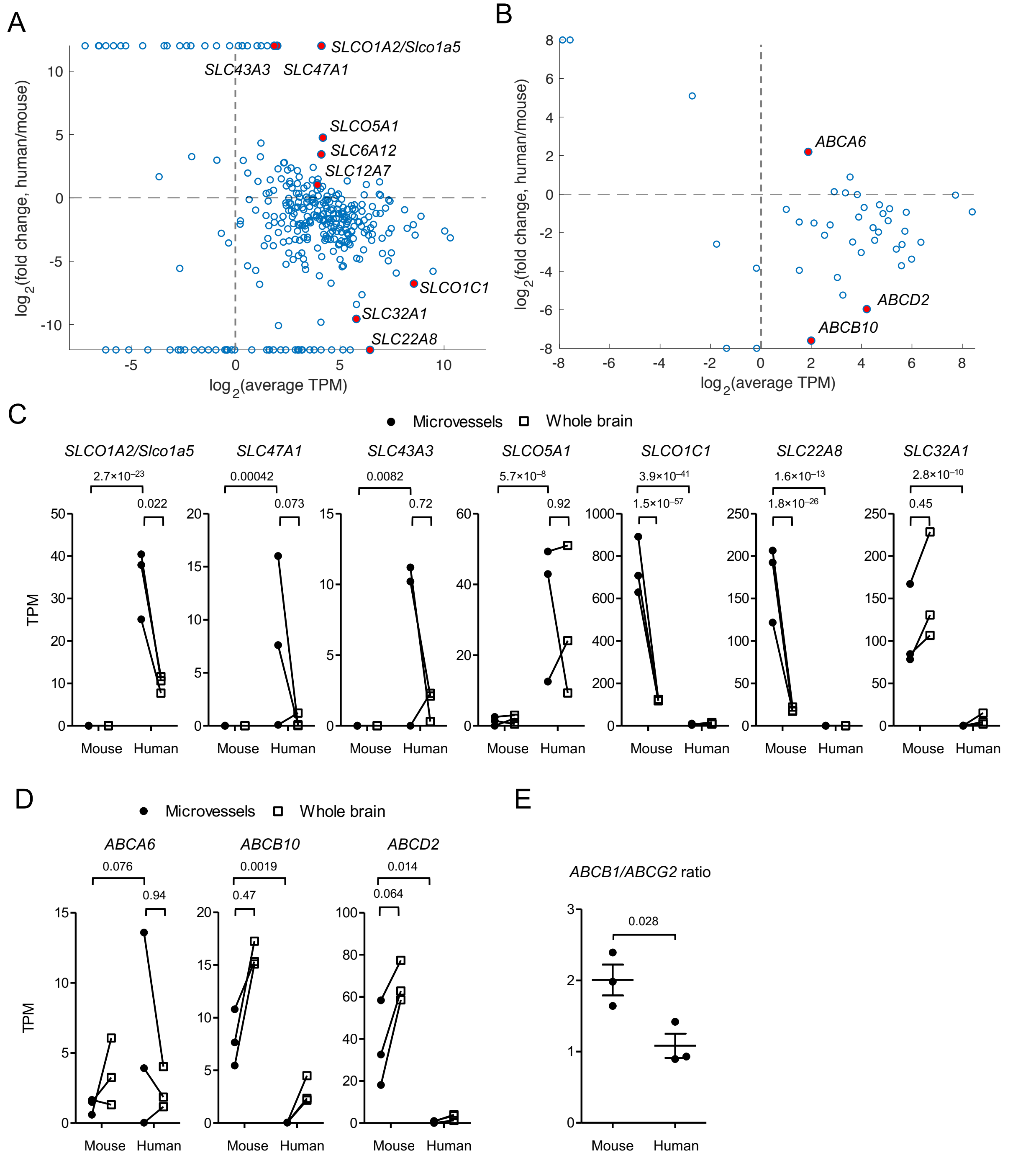

Figure S6. Mouse-human species differences in *SLC* and *ABC* transporter expression. (A) Comparison of solute carrier (*SLC*) transcript abundance in human and mouse LCM microvessels. The $\log_2$(fold change) of human transcript abundance versus the homologous mouse transcript abundance is plotted against the average abundance in human and mouse. Each

point represents one gene with known mouse-human homology. Genes with zero expression in all samples were excluded. Genes to the right of the vertical line at $\log_2$(average TPM) = 0 are moderately or highly expressed in at least one species. A subset of human-enriched *SLC*s (*SLCO1A2*, *SLC47A1*, *SLC43A3*, *SLCO5A1*, *SLC6A12*, and *SLC12A7*) and mouse-enriched *SLC*s (*SLCO1C1*, *SLC22A8*, and *SLC32A1*) are highlighted. Genes with zero expression in one species were visualized with an average $\log_2$(fold change) of ± 12. Complete results of this analysis are in Supplementary Table S5. **(B)** Comparison of ATP binding cassette (*ABC*) transcript abundance in human and mouse LCM microvessels. The $\log_2$(fold change) of human transcript abundance versus the homologous mouse transcript abundance is plotted against the average abundance in human and mouse. Each point represents one gene with known mouse-human homology. Genes with zero expression in all samples were excluded. Genes to the right of the vertical line at $\log_2$(average TPM) = 0 are moderately or highly expressed in at least one species. A subset of human-enriched *ABC*s (*ABCA6*) and mouse-enriched *ABC*s (*ABCB10* and *ABCD2*) are highlighted. Genes with zero expression in one species were visualized with an average $\log_2$(fold change) of ± 8. Complete results of this analysis are in Supplementary Table S5. **(C)** Transcript abundance of selected genes in biological triplicates of LCM microvessels and whole brain from human and mouse. *Slco1a5* is the mouse homolog of human *SLCO1A2*. Lines connect datapoints from matched LCM microvessel and whole brain samples. Adjusted P-values (from DESeq2) are shown; Wald test with Benjamini-Hochberg correction. **(D)** Transcript abundance of genes highlighted in (B) in biological triplicates of LCM microvessels and whole brain from human and mouse. Lines connect datapoints from matched LCM microvessel and whole brain samples. Adjusted P-values (from DESeq2) are shown; Wald test with Benjamini-Hochberg correction. **(E)** Ratio of *ABCB1* (mouse *Abcb1a*) to *ABCG2* transcript abundance (TPM) in human and mouse LCM microvessels. P-value shown from Student's *t* test.

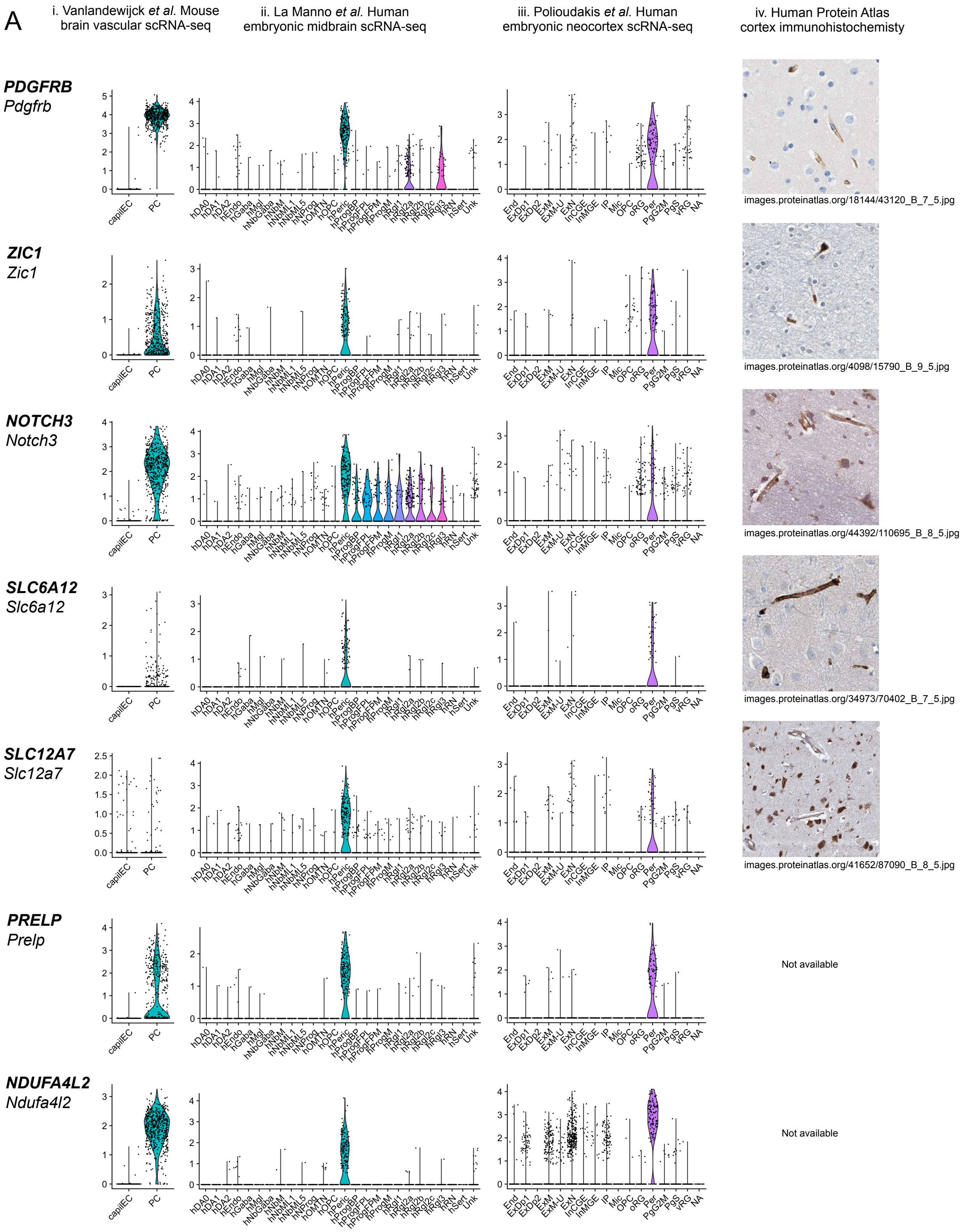
A
i. Vanlandewijck *et al.* Mouse brain vascular scRNA-seq
ii. La Manno *et al.* Human embryonic midbrain scRNA-seq
iii. Polioudakis *et al.* Human embryonic neocortex scRNA-seq
iv. Human Protein Atlas cortex immunohistochemistry
PDGFRB *Pdgfrb*
images.proteinatlas.org/18144/43120_B_7_5.jpg
ZIC1 *Zic1*
images.proteinatlas.org/4098/15790_B_9_5.jpg
NOTCH3 *Notch3*
images.proteinatlas.org/44392/110695_B_8_5.jpg
SLC6A12 *Slc6a12*
images.proteinatlas.org/34973/70402_B_7_5.jpg
SLC12A7 *Slc12a7*
images.proteinatlas.org/41652/87090_B_8_5.jpg
PRELP *Prelp*
Not available
NDUFA4L2 *Ndufa4l2*
Not available
capilEC PC
hDA0 hDA1 hDA2 hEndo hGaba hMgl hNbGaba hNbM hNbML1 hNbML5 hNProg hOMTN hOPC hPeric hProgBP hProgFPL hProgFPM hProgM hRgl1 hRgl2a hRgl2b hRgl2c hRgl3 hRN hSert Unk
End ExDp1 ExDp2 ExM ExM-U ExN InCGE InMGE IP Mic OPC oRG Per PgG2M PgS vRG NA

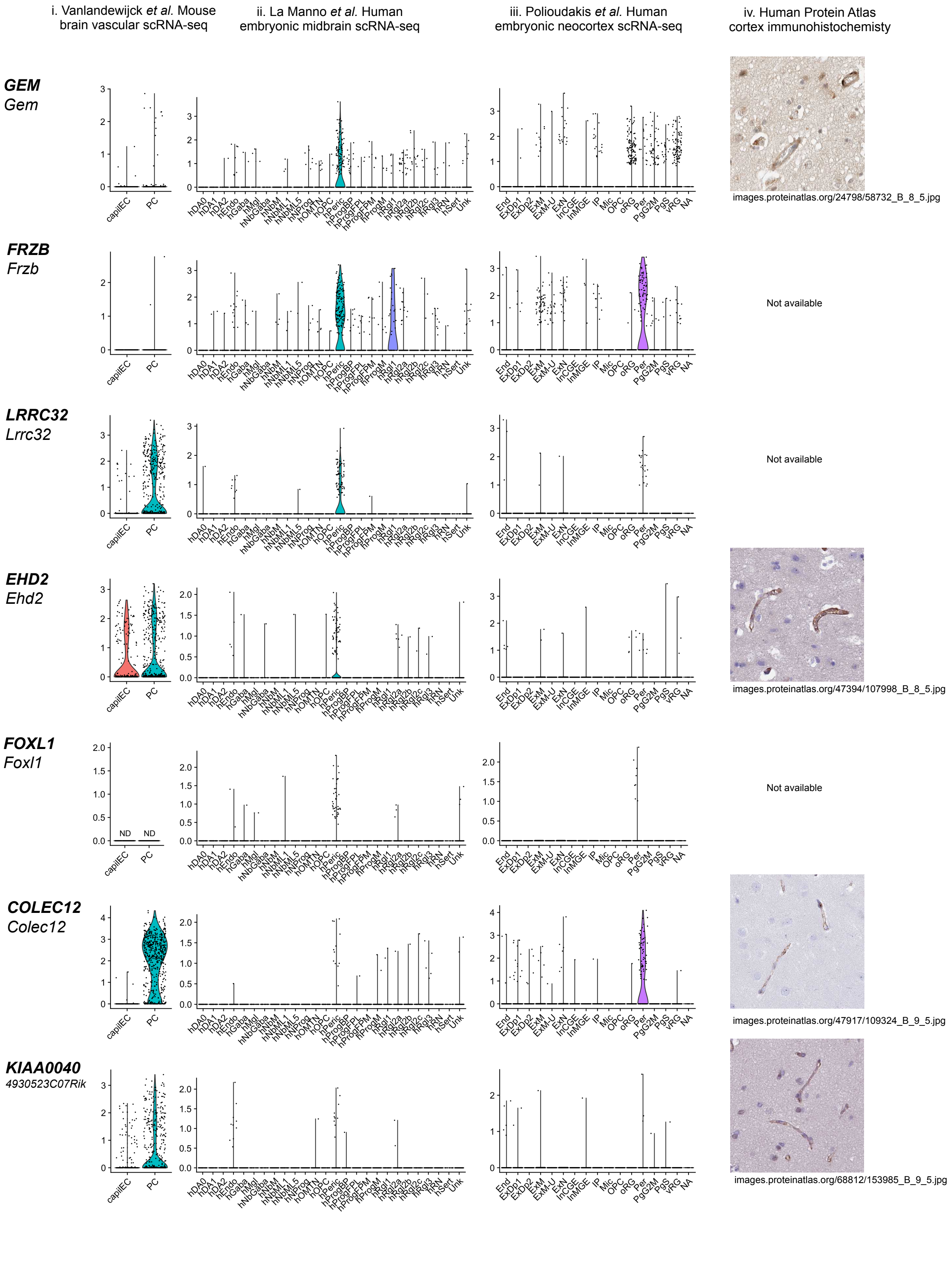
i. Vanlandewijck et al. Mouse brain vascular scRNA-seq
ii. La Manno et al. Human embryonic midbrain scRNA-seq
iii. Polioudakis et al. Human embryonic neocortex scRNA-seq
iv. Human Protein Atlas cortex immunohistochemistry
GEM
Gem
images.proteinatlas.org/24798/58732_B_8_5.jpg
FRZB
Frzb
Not available
LRRC32
Lrrc32
Not available
EHD2
Ehd2
images.proteinatlas.org/47394/107998_B_8_5.jpg
FOXL1
Foxl1
ND
ND
Not available
COLEC12
Colec12
images.proteinatlas.org/47917/109324_B_9_5.jpg
KIAA0040
4930523C07Rik
images.proteinatlas.org/68812/153985_B_9_5.jpg
capilEC
PC
hDA0
hDA1
hDA2
hEndo
hGaba
hMgl
hNbGaba
hNbM
hNbML1
hNbML5
hNProg
hOMTN
hOPC
hPeric
hProgBP
hProgFPL
hProgFPM
hProgM
hRgl1
hRgl2a
hRgl2b
hRgl2c
hRgl3
hRN
hSert
Unk
End
ExDp1
ExDp2
ExM
ExM-U
ExN
InCGE
InMGE
IP
Mic
OPC
oRG
Per
PgG2M
PgS
vRG
NA

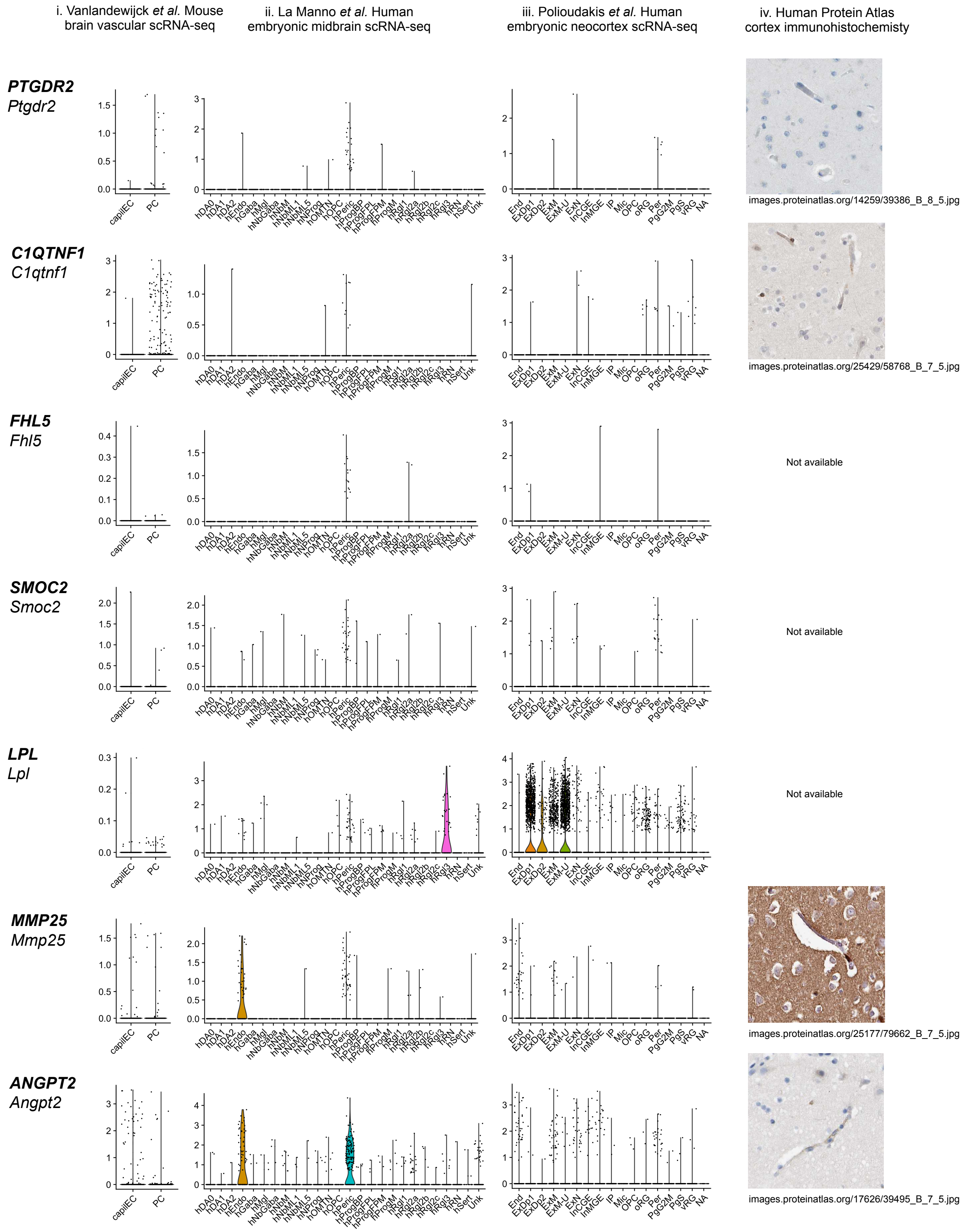
i. Vanlandewijck et al. Mouse brain vascular scRNA-seq
ii. La Manno et al. Human embryonic midbrain scRNA-seq
iii. Polioudakis et al. Human embryonic neocortex scRNA-seq
iv. Human Protein Atlas cortex immunohistochemisty
PTGDR2
Ptgdr2
images.proteinatlas.org/14259/39386_B_8_5.jpg
C1QTNF1
C1qtnf1
images.proteinatlas.org/25429/58768_B_7_5.jpg
FHL5
Fhl5
Not available
SMOC2
Smoc2
Not available
LPL
Lpl
Not available
MMP25
Mmp25
images.proteinatlas.org/25177/79662_B_7_5.jpg
ANGPT2
Angpt2
images.proteinatlas.org/17626/39495_B_7_5.jpg
capilEC
PC
hDA0
hDA1
hDA2
hEndo
hGaba
hMgl
hNbGaba
hNbM
hNbML1
hNbML5
hNProg
hOMTN
hOPC
hPeric
hProgBP
hProgFPL
hProgFPM
hProgM
hRgl1
hRgl2a
hRgl2b
hRgl2c
hRgl3
hRN
hSert
Unk
End
ExDp1
ExDp2
ExM
ExM-U
ExN
InCGE
InMGE
IP
Mic
OPC
oRG
Per
PgG2M
PgS
vRG
NA

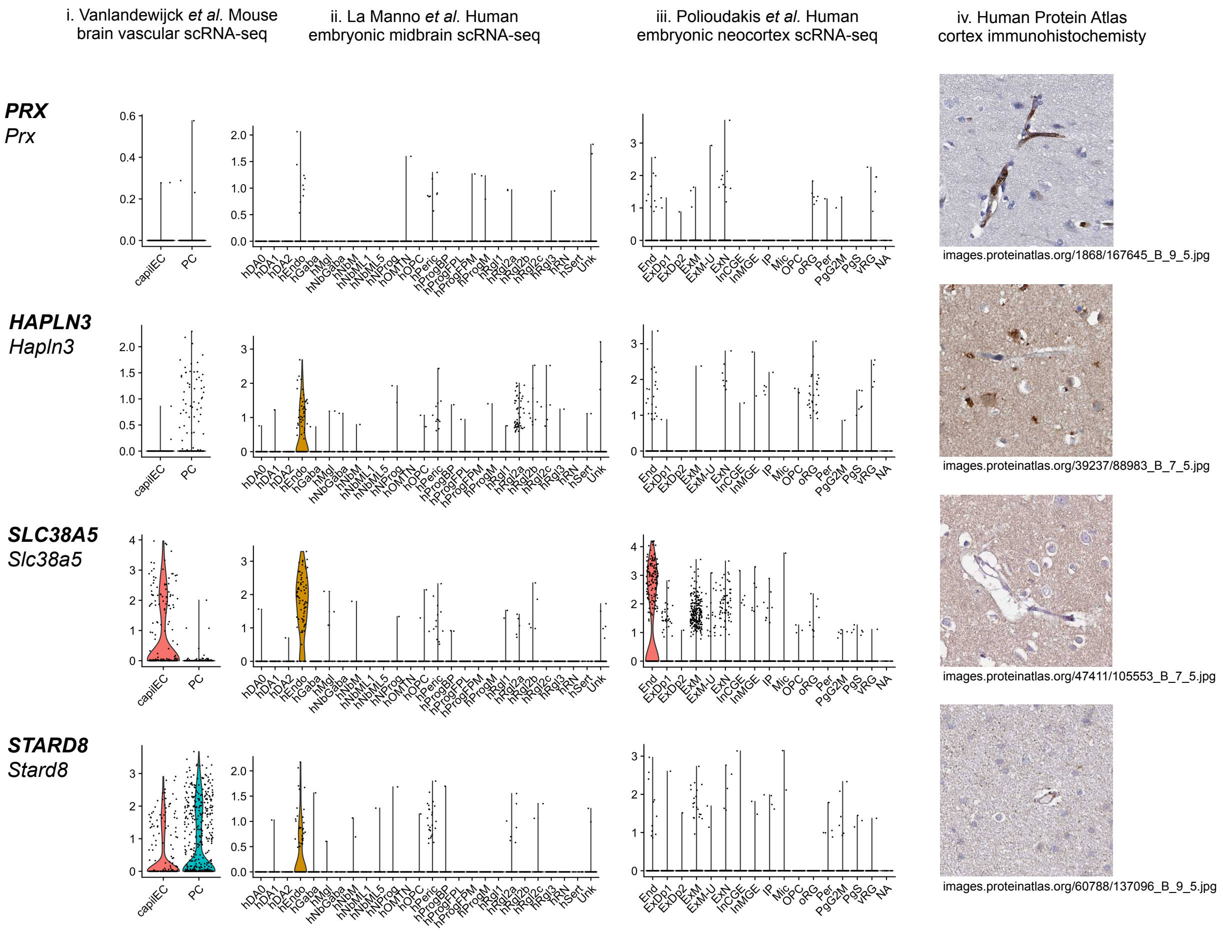
i. Vanlandewijck et al. Mouse brain vascular scRNA-seq
ii. La Manno et al. Human embryonic midbrain scRNA-seq
iii. Polioudakis et al. Human embryonic neocortex scRNA-seq
iv. Human Protein Atlas cortex immunohistochemisty
PRX
Prx
images.proteinatlas.org/1868/167645_B_9_5.jpg
HAPLN3
Hapln3
images.proteinatlas.org/39237/88983_B_7_5.jpg
SLC38A5
Slc38a5
images.proteinatlas.org/47411/105553_B_7_5.jpg
STARD8
Stard8
images.proteinatlas.org/60788/137096_B_9_5.jpg
capilEC
PC
End
ExDp1
ExDp2
ExM
ExM-U
ExN
InCGE
InMGE
IP
Mic
OPC
oRG
Per
PgG2M
PgS
vRG
NA

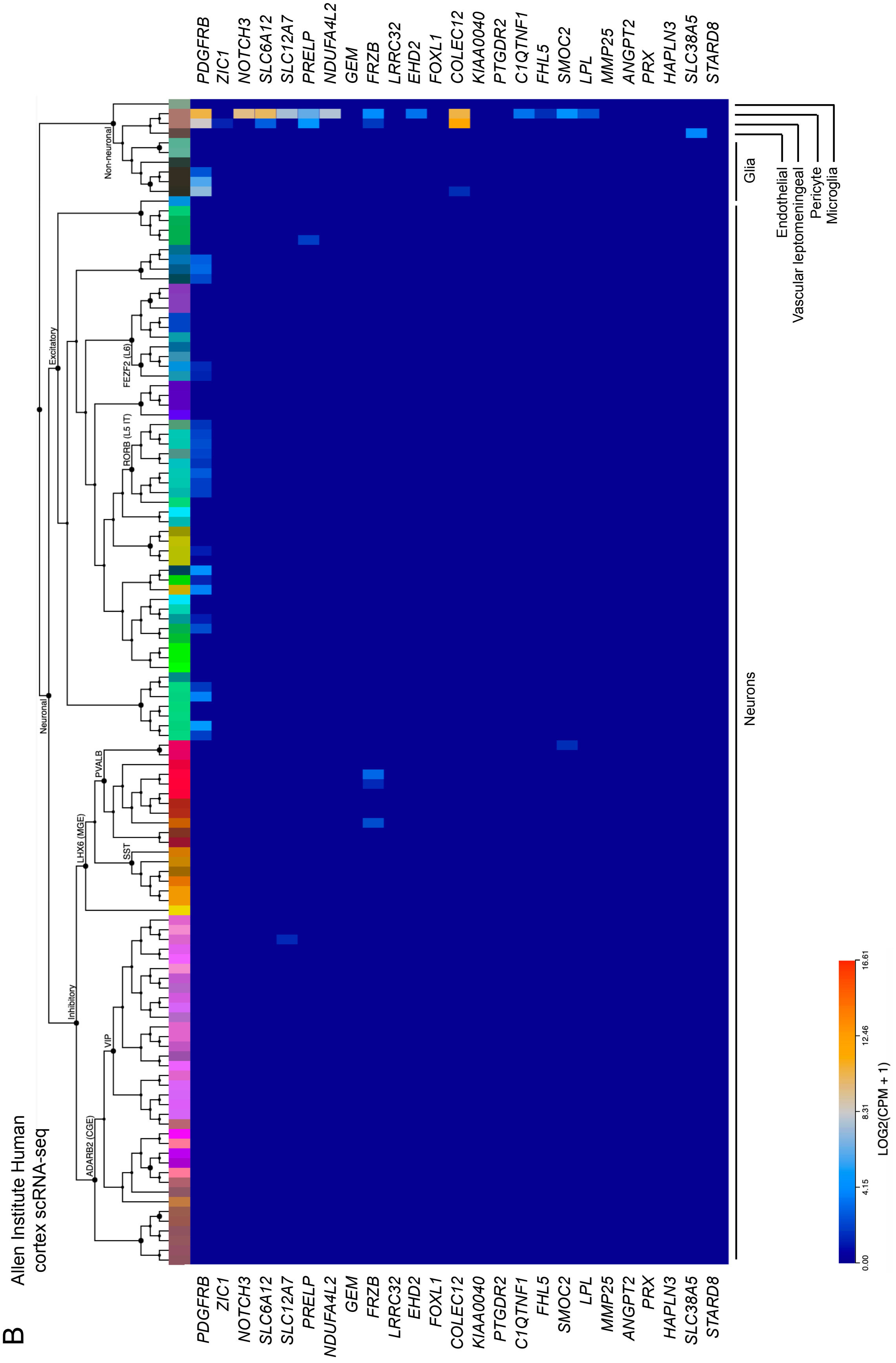
B
Allen Institute Human
cortex scRNA-seq
Neuronal
Inhibitory
Excitatory
Non-neuronal
ADARB2 (CGE)
VIP
LHX6 (MGE)
SST
PVALB
RORB (L5 IT)
FEZF2 (L6)
PDGFRB
ZIC1
NOTCH3
SLC6A12
SLC12A7
PRELP
NDUFA4L2
GEM
FRZB
LRRC32
EHD2
FOXL1
COLEC12
KIAA0040
PTGDR2
C1QTNF1
FHL5
SMOC2
LPL
MMP25
ANGPT2
PRX
HAPLN3
SLC38A5
STARD8
Neurons
Glia
Endothelial
Vascular leptomeningeal
Pericyte
Microglia
LOG2(CPM + 1)
0.00
4.15
8.31
12.46
16.61

Figure S7. Validation of putative human brain pericyte-enriched transcripts. (A) Each row shows data from several sources for one transcript/protein of interest. (i) Transcript abundance (counts) in mouse brain pericytes and capillary endothelial cells [9,10]. Cell assignment to clusters and cluster names are as reported by Vanlandewjick *et al.* [9,10]. (ii) Transcript abundance (counts) in human embryonic midbrain single cell RNA-seq data [18]. Cell assignment to clusters and cluster names are as reported by La Manno *et al.* [18]. hPeric: human pericytes. hEndo: human endothelial cells. (iii) Transcript abundance (counts) in human embryonic neocortex single cell RNA-seq data [19]. Cell assignment to clusters and cluster names are as reported by Polioudakis *et al.* [19]. Per: pericytes. End: endothelial cells. (iv) Immunohistochemistry on human cortex samples from the Human Protein Atlas (v19.proteinatlas.org) [32]. URLs for each image are provided in the figure. **(B)** Heat map of transcript abundance in human cortex single cell and single nucleus RNA-seq [39]. Figure adapted from http://celltypes.brain-map.org/rnaseq/human/cortex.

Table S1. Characteristics of RNA samples and associated RNA-seq datasets

Sample	Number collected	Total surface area (mm^2)	Total RNA (ng)	Total reads	Uniquely mapped reads (%)	Multi-mapping reads (%)
Mouse microvessels 1	1839	2.2	1.0	19,703,651	82.95	9.75
Mouse microvessels 2	3058	3.0	3.1	22,370,744	83.48	11.67
Mouse microvessels 3	2841	2.5	1.0	21,081,514	85.02	10.73
Mouse whole brain 1	28	2.1	2.2	19,617,678	88.88	8.69
Mouse whole brain 2	22	3.0	4.4	19,500,744	80.91	9.23
Mouse whole brain 3	21	2.4	4.4	19,206,425	88.96	8.56
Human microvessels 1	2004	2.3	0.8	27,757,265	58.47	21.82
Human microvessels 2	2577	3.0	4.6	27,762,525	43.84	33.75
Human microvessels 3	2200	2.3	0.9	27,161,926	56.96	24.01
Human whole brain 1	27	2.3	5.5	24,842,174	59.55	22.43
Human whole brain 2	34	3.0	4.7	33,073,030	51.88	28.52
Human whole brain 3	19	2.3	1.5	30,941,286	65.76	15.41
